# Author Correction: Inactivated vaccine-elicited potent antibodies can broadly neutralize SARS-CoV-2 circulating variants

**DOI:** 10.1038/s41467-026-79108-1

**Published:** 2026-10-30

**Authors:** Yubin Liu, Ziyi Wang, Xinyu Zhuang, Shengnan Zhang, Zhicheng Chen, Yan Zou, Jie Sheng, Tianpeng Li, Wanbo Tai, Jinfang Yu, Yanqun Wang, Zhaoyong Zhang, Yunfeng Chen, Liangqin Tong, Xi Yu, Linjuan Wu, Dong Chen, Renli Zhang, Ningyi Jin, Weijun Shen, Jincun Zhao, Mingyao Tian, Xinquan Wang, Gong Cheng

**Affiliations:** 1 Tsinghua-Peking Joint Center for Life Sciences, School of Medicine, Tsinghua University, Beijing, 100084 China; 2 Institute of Infectious Diseases, Shenzhen Bay Laboratory, Shenzhen Guangdong, 518132 China; 3 The Ministry of Education Key Laboratory of Protein Science, Beijing Advanced Innovation Center for Structural Biology, Beijing Frontier Research Center for Biological Structure, Collaborative Innovation Center for Biotherapy, School of Life Sciences, Tsinghua University, Beijing, 100084 China; 4 Changchun Veterinary Research Institute, Chinese Academy of Agricultural Sciences, Changchun, 130122 China; 5 State Key Laboratory of Respiratory Disease, Guangzhou Institute of Respiratory Disease, the First Affiliated Hospital of Guangzhou Medical University, Guangzhou, 510182 China; 6 Center for Translational Research, Shenzhen Bay Laboratory, Shenzhen Guangdong, 518132 China; 7 Wenzhou Central Hospital, Wenzhou, 325000 China; 8 Institute of Pathogenic Organisms, Shenzhen Center for Disease Control and Prevention, Shenzhen Guangdong, 518055 China

**Keywords:** Immunology, Inactivated vaccines, SARS-CoV-2, Antibodies

Correction to: *Nature Communications* https://doi.org/10.1038/s41467-023-37926-7, published online 17 April 2023

In the original version of this article, the SARS-CoV-2 variant name “BF.11” in the Introduction was incorrect and should have read “BF.7”.

In addition, due to errors during Source Data compilation, several incorrect entries were included in the Source Data associated with Figs. 2d and 3e. The corresponding Source Data, Figs. 2d and 3e, and neutralization curves in Supplementary Figs. 5 and 12 have been corrected where required.

These corrections do not affect the interpretation, main findings or conclusions of the article. The text, figures, and Source Data are now updated in the HTML and PDF versions of the article. For comparison, the original figures are available as Supplementary Information accompanying this amendment.

## Supplementary information

Original Figs. 2, 3, Supplementary Figs. 5, 12

