# Supplementary material for: Inactivated vaccine-elicited potent antibodies can broadly neutralize SARS-CoV-2 circulating variants
Source: Nat Commun. 2023 Apr 17;14:2179. doi: 10.1038/s41467-023-37926-7 (PMC10107573; doi:10.1038/s41467-023-37926-7)
Supplement: Supplementary file 1 — Supplementary Information [file 41467_2023_37926_MOESM1_ESM.pdf]

Supplementary Materials for

Inactivated vaccine-elicited potent antibodies can broadly neutralize SARS-CoV-2 circulating variants

Yubin Liu, Ziyi Wang, Xinyu Zhuang, Shengnan Zhang, Zhicheng Chen, Yan Zou, Jie Sheng, Tianpeng Li, Wanbo Tai, Jinfang Yu, Yanqun Wang, Zhaoyong Zhang, Yunfeng Chen, Liangqin Tong, Xi Yu, Linjuan Wu, Dong Chen, Renli Zhang, Ningyi Jin, Weijun Shen, Jincun Zhao, Mingyao Tian, Xinquan Wang, Gong Cheng

*Corresponding to

wshen@szbl.ac.cn

zhaojincun@gird.cn;

klwklw@126.com;

xinquanwang@mail.tsinghua.edu.cn;

gongcheng@mail.tsinghua.edu.cn.

This file includes:

Supplementary Fig. 1 Plasma antibody response to SARS-CoV-2 vaccination.

Supplementary Fig. 2 Kinetics of SARS-CoV-2 spike-biding memory B-cell responses.

Supplementary Fig. 3 Isolation of SARS-CoV-2 RBD-specific antibodies.

Supplementary Fig. 4 Frequency distributions of human V genes in vaccinated individuals.

Supplementary Fig. 5 Antibody neutralization analyzed with pseudovirus SARS-CoV-2 wild type and variants.

Supplementary Fig. 6 Epitope mapping through competitive binding measured by biolayer interferometry (BLI).

Supplementary Fig. 7 Competitive binding of monoclonal antibody 6-2C with 10-5B and 13-1C measured by BLI.

Supplementary Fig. 8 Antibody and hACE2 competition for binding to SARS-CoV-2 RBD determined by BLI.

Supplementary Fig. 9 Antibody neutralization analyzed with authentic SARS-CoV-2 Beta and Delta variants.

Supplementary Fig. 10 SDS-PAGE analysis of four bispecific antibodies BI-2C5B, BI-5B2C, BI-2C1C and BI-1C2C.

Supplementary Fig. 11 Binding analysis of monoclonal antibodies with SARS-CoV-2 RBDs measured by BLI.

Supplementary Fig. 12 Bispecific antibodies neutralization analyzed with pseudovirus SARS-CoV-2 wild type and variants.

Supplementary Fig. 13 Cryo-EM data processing for wild-type S trimer in complex with10-5B.

Supplementary Fig. 14 Cryo-EM data processing for Omicron BA.1 S trimer in complex with 10-5B and 6-2C.

Supplementary Fig. 15 Cryo-EM data processing for Omicron BA.4 S trimer in complex with 10-5B and 6-2C.

Supplementary Fig. 16 The Cryo-EM density maps of antibody-RBD interface.

Supplementary Fig. 17 Structural characterization of mAbs 6-2C and 10-5B.

Supplementary Fig. 18 The loop with residues 371–377 in the spike of Omicron BA.1 and BA.4.

Supplementary Fig. 19 Superimposition of 10-5B-bound, 6-2C-bound, and ACE2-bound [PDB: 6M0J] SARS-CoV-2 RBD structures.

Supplementary Fig. 20 Structural characterization of the bsAb BI-2C5B.

Supplementary Fig. 21 Plasma antibodies specific for different epitopes on SARS-CoV-2 RBD measured by competitive ELISA.

Supplementary Table 1 Characteristics of the participants.

Supplementary Table 2 SARS-CoV-2 variants tested in this study.

Supplementary Table 3 Characteristics of subjects selected for antibody isolation.

Supplementary Table 4 Molecular characteristics of neutralizing antibodies.

Supplementary Table 5 Neutralization profile of monoclonal antibodies against SARS-CoV-2 variants.

Supplementary Table 6 Crystallization data collection and refinement statistics.

Supplementary Table 7 Cryo-EM data collection, refinement, and validation statistics.

Supplementary Table 8 Characteristics of subjects with high percentages of Spike-specific memory B cells after the booster dose.

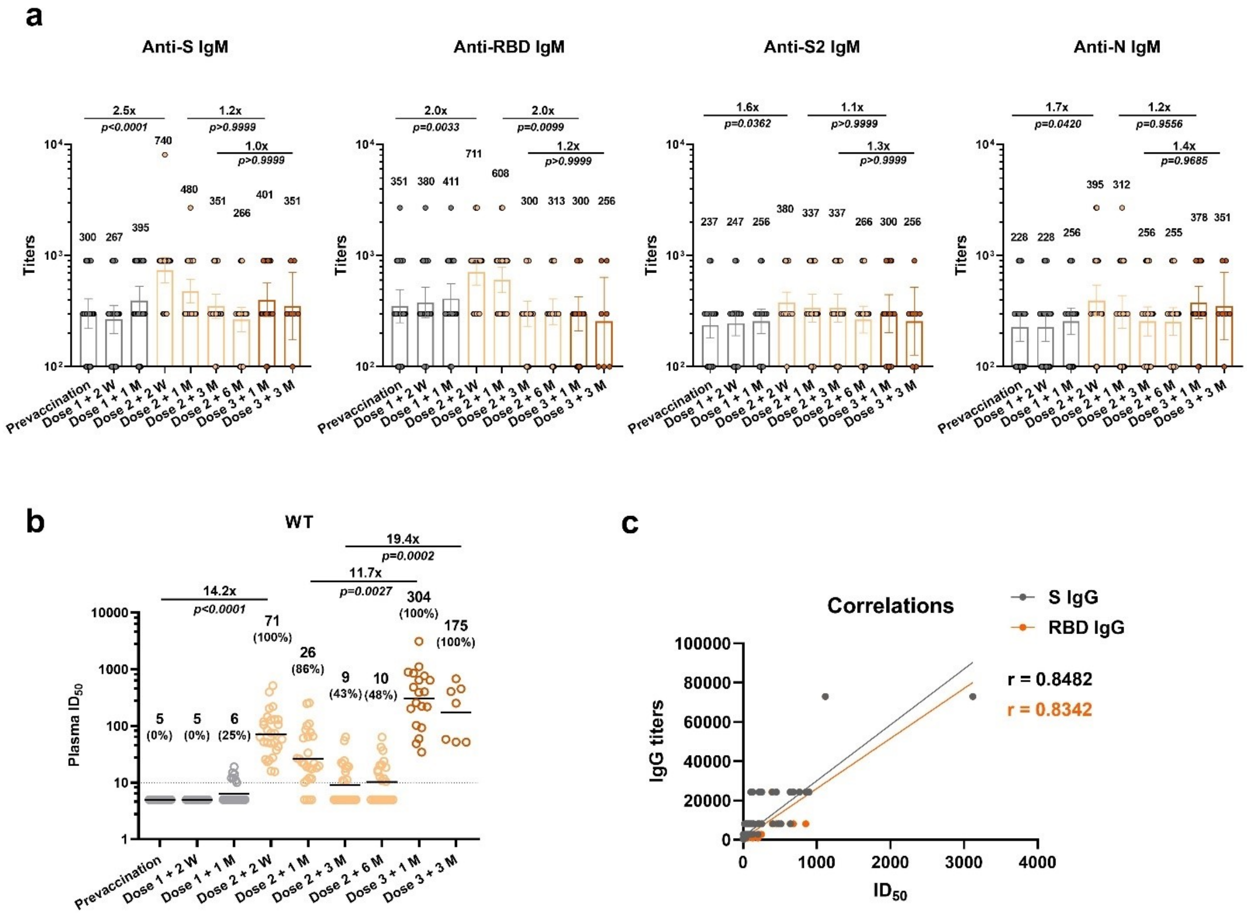

Supplementary Fig. 1 Plasma antibody response to SARS-CoV-2 vaccination.

(a) Longitudinal plasma IgM titers to the spike (S), receptor-binding-domain (RBD), S2 region of spike, and nucleocapsid (N) proteins measured by ELISA. Samples were collected before immunization (Prevaccination, n = 28), at 2 weeks (Dose 1 + 2 W, n=28) and 1 month (Dose 1 + 1 M, n=28) after the first dose, at 2 weeks (Dose 2 + 2 W, n=28), 1 month (Dose 2 + 1 M, n=28), 3 months (Dose 2 + 3 M, n=28) and 6 months (Dose 2 + 6 M, n=27) after the second dose, and at a median of 1 month (Dose 3 + 1 M, n=19) and 3 months (Dose 3 + 1 M, n=7) after the third dose (for details see Supplementary Table 1). Data are shown as geometric mean titers (GMTs) (values above bars) with 95% confidence interval (CI). The lower limit of quantification (LLOQ) was 100 for IgM titers. P values were determined using two-sided Kruskal–Wallis test with subsequent Dunn's multiple comparisons.

(b) Longitudinal plasma neutralizing activity against WT SARS-CoV-2 as in **a**. The 50% inhibitory dilution (ID_{50}) neutralizing antibody titers were determined by pseudovirus neutralization assays. Black bars and values above points represent the geometric mean ID_{50},

and the percentages of samples with detectable neutralizing activity above the lower limit of quantification (LLOQ) are given in parentheses. The horizontal dashed lines indicate the LLOQ. For values below the LLOQ, LLOD/2 values were plotted. The LLOQ of the assay for ID_{50} was 10. P values were determined using two-sided Kruskal–Wallis test with subsequent Dunn's multiple comparisons.

(c) Correlations of plasma 50% inhibitory dilution (ID_{50}) neutralizing antibody titers to WT SARS-CoV-2 with anti-S and anti-RBD IgG titers (n=221). The r and p values were determined by two-tailed Spearman's correlations.

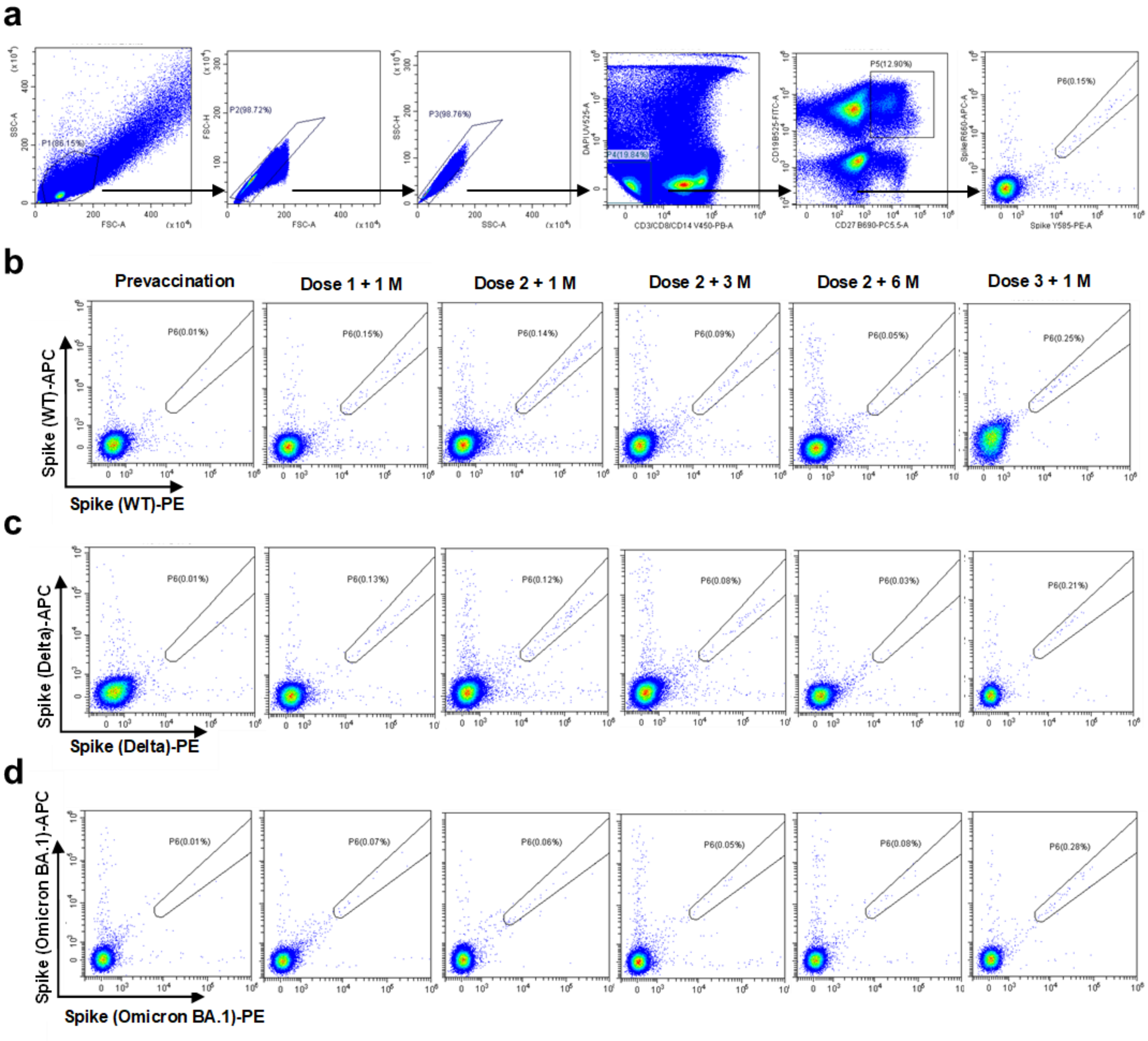

Supplementary Fig. 2 Kinetics of SARS-CoV-2 spike-binding memory B-cell responses.

(**a**) Gating strategy used for cell analysis. Gating was performed on singlets that were $CD3^-CD8^-CD14^-CD19^+CD27^+$ Spike-PE^+ and Spike-APC^+.

(**b-d**) Representative flow cytometry plots showing dual Spike-PE- and Spike-APC-binding B cells against SARS-CoV-2 wild type (WT) (**b**), Delta (**c**) and Omicron BA.1 (**d**) for a vaccinated individual.

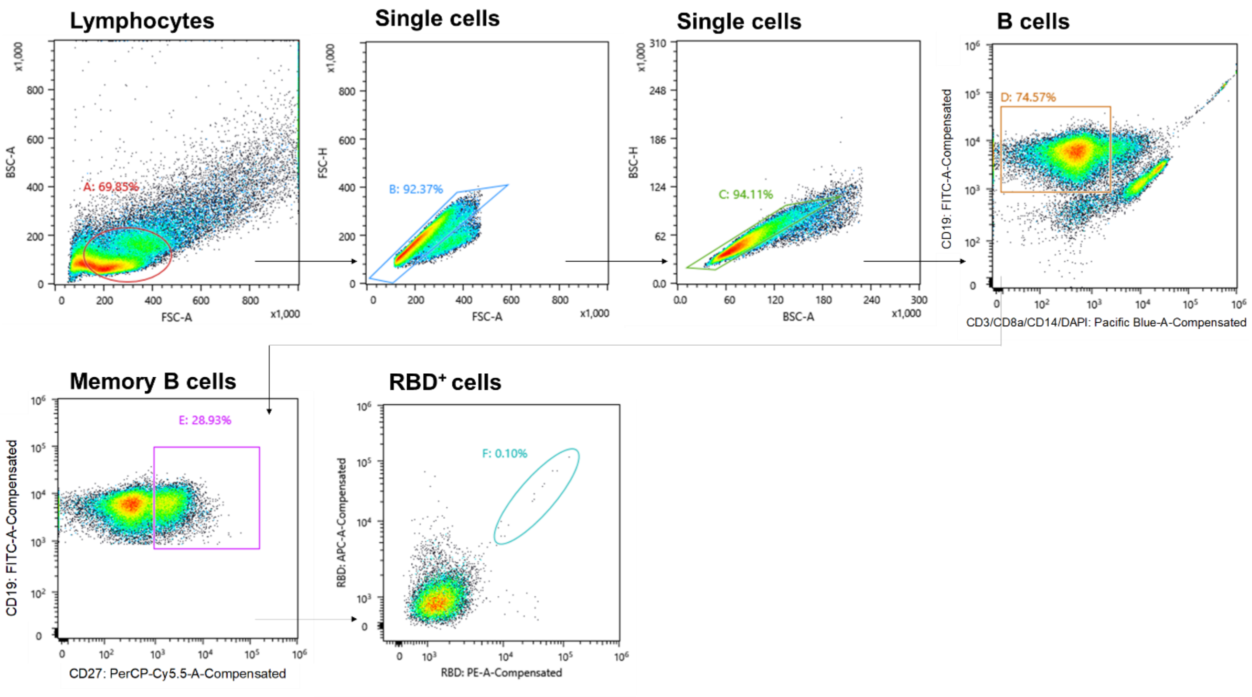

Supplementary Fig. 3 Isolation of SARS-CoV-2 RBD-specific antibodies. Gating strategy for the isolation of RBD-specific memory B cells through fluorescence-activated cell sorting (FACS). Lymphocytes were gated on all the loading cells, followed by two selections of single cells. Then B cells ($CD3^-CD8^-CD14^-CD19^+$) gating were on singlets. Finally, memory B cells ($CD19^+CD27^+$) were gated, from which RBD-PE^+ and RBD-APC^+ cells were sorted. FSC-A: forward scatter area. FSC-H: forward scatter height. BSC-A: back scatter area. BSC-H: back scatter height.

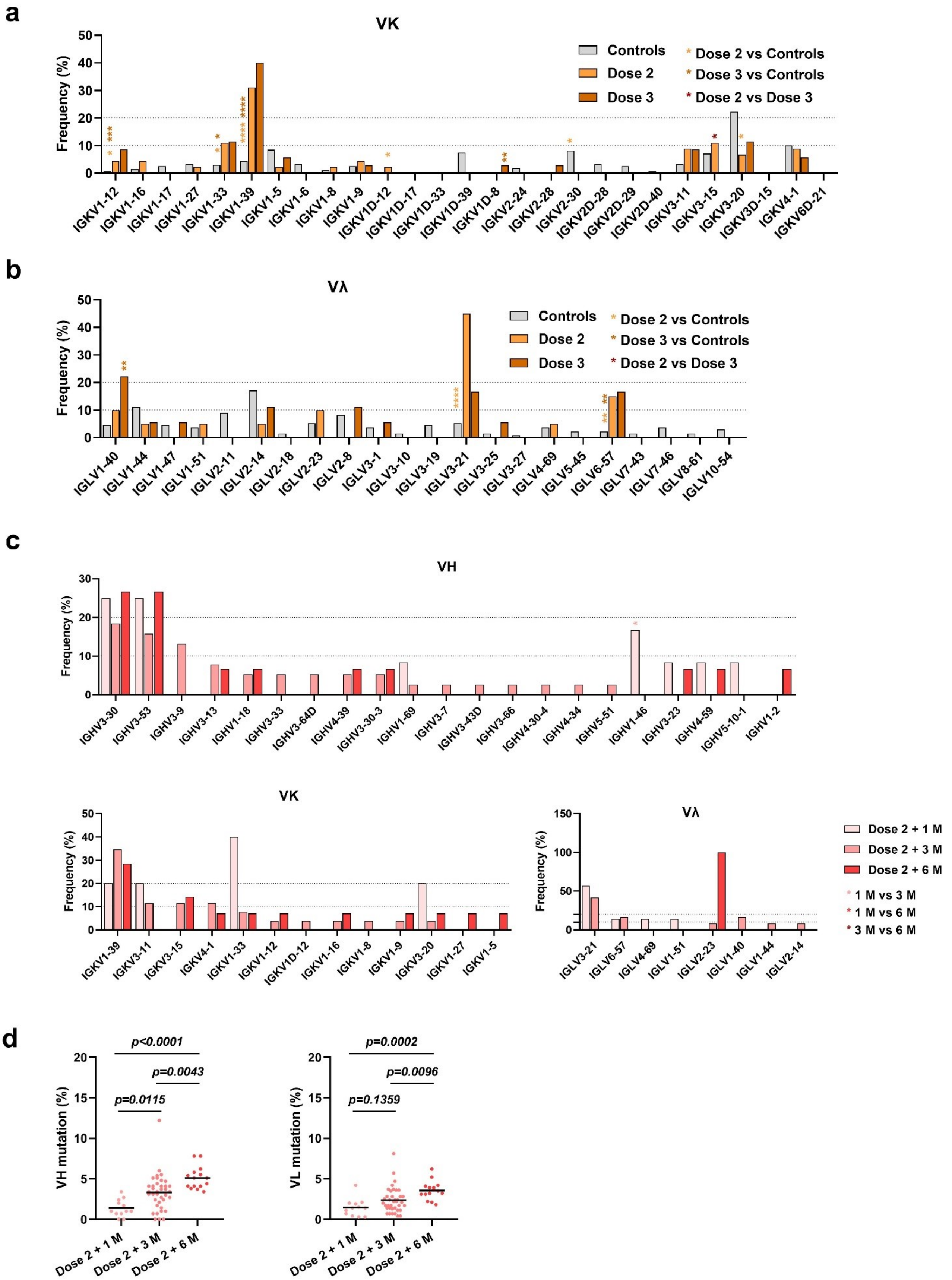
a
VK
Frequency (%)
Controls
Dose 2
Dose 3
* Dose 2 vs Controls
* Dose 3 vs Controls
* Dose 2 vs Dose 3
IGKV1-12 IGKV1-16 IGKV1-17 IGKV1-27 IGKV1-33 IGKV1-39 IGKV1-5 IGKV1-6 IGKV1-8 IGKV1-9 IGKV1D-12 IGKV1D-17 IGKV1D-33 IGKV1D-39 IGKV1D-8 IGKV2-24 IGKV2-28 IGKV2-30 IGKV2D-28 IGKV2D-29 IGKV2D-40 IGKV3-11 IGKV3-15 IGKV3-20 IGKV3D-15 IGKV4-1 IGKV6D-21
b
Vλ
Frequency (%)
Controls
Dose 2
Dose 3
* Dose 2 vs Controls
* Dose 3 vs Controls
* Dose 2 vs Dose 3
IGLV1-40 IGLV1-44 IGLV1-47 IGLV1-51 IGLV2-11 IGLV2-14 IGLV2-18 IGLV2-23 IGLV2-8 IGLV3-1 IGLV3-10 IGLV3-19 IGLV3-21 IGLV3-25 IGLV3-27 IGLV4-69 IGLV5-45 IGLV6-57 IGLV7-43 IGLV7-46 IGLV8-61 IGLV10-54
c
VH
Frequency (%)
IGHV3-30 IGHV3-53 IGHV3-9 IGHV3-13 IGHV1-18 IGHV3-33 IGHV3-64D IGHV4-39 IGHV3-30-3 IGHV1-69 IGHV3-7 IGHV3-43D IGHV3-66 IGHV4-30-4 IGHV4-34 IGHV5-51 IGHV1-46 IGHV3-23 IGHV4-59 IGHV5-10-1 IGHV1-2
VK
Frequency (%)
IGKV1-39 IGKV3-11 IGKV3-15 IGKV4-1 IGKV1-33 IGKV1-12 IGKV1D-12 IGKV1-16 IGKV1-8 IGKV1-9 IGKV3-20 IGKV1-27 IGKV1-5
Vλ
Frequency (%)
IGLV3-21 IGLV6-57 IGLV4-69 IGLV1-51 IGLV2-23 IGLV1-40 IGLV1-44 IGLV2-14
Dose 2 + 1 M
Dose 2 + 3 M
Dose 2 + 6 M
* 1 M vs 3 M
* 1 M vs 6 M
* 3 M vs 6 M
d
VH mutation (%)
p<0.0001
p=0.0043
p=0.0115
Dose 2 + 1 M
Dose 2 + 3 M
Dose 2 + 6 M
VL mutation (%)
p=0.0002
p=0.0096
p=0.1359
Dose 2 + 1 M
Dose 2 + 3 M
Dose 2 + 6 M

Supplementary Fig. 4 Characteristics of V genes in anti-SARS-CoV-2 RBD monoclonal antibodies in vaccinated individuals.

(**a** and **b)** The graph shows the relative abundance of human IGVK (**a**) and IGVL (**b**) genes of anti-SARS-CoV-2 RBD monoclonal antibodies (mAbs) (n=118) from vaccinees, compared to those in IgG-expressing memory B repertoires (n=403) of healthy human donors. The mAbs were isolated from PBMCs collected 1 month, 3 months and 6 months after the second vaccine dose and 1 month after the third dose. Control group data were obtained from three prevaccinated samples using single cell sequencing as described in the methods. Statistical significance was determined by two-sided Chi-square test with 1 degree of freedom (* $p < 0.05$, ** $p < 0.01$, *** $p < 0.001$, **** $p < 0.0001$).

(**c**) The frequency distribution of human IGVH, IGVK and IGVL of anti-SARS-CoV-2 RBD monoclonal antibodies (mAbs) from PBMCs sampled 1 month (Dose 2 + 1 M, n=12), 3 months (Dose 2 + 3 M, n=38) and 6 months (Dose 2 + 6 M, n=15) after the second vaccine dose (Dose 2). Statistical significance was determined by two-sided Chi-square test with 1 degree of freedom (* $p < 0.05$, ** $p < 0.01$, *** $p < 0.001$, **** $p < 0.0001$).

(**d**) The nucleotide somatic hypermutation levels of the V region in the heavy chain and light chain from samples obtained 1 month (Dose 2 + 1 M, n=12), 3 months (Dose 2 + 3 M, n=38) and 6 months (Dose 2 + 6 M, n=15) after the second vaccine dose (Dose 2). The horizontal bars indicate the mean values. Statistical significance was determined by two-sided Kruskal–Wallis test with subsequent Dunn's multiple comparisons.

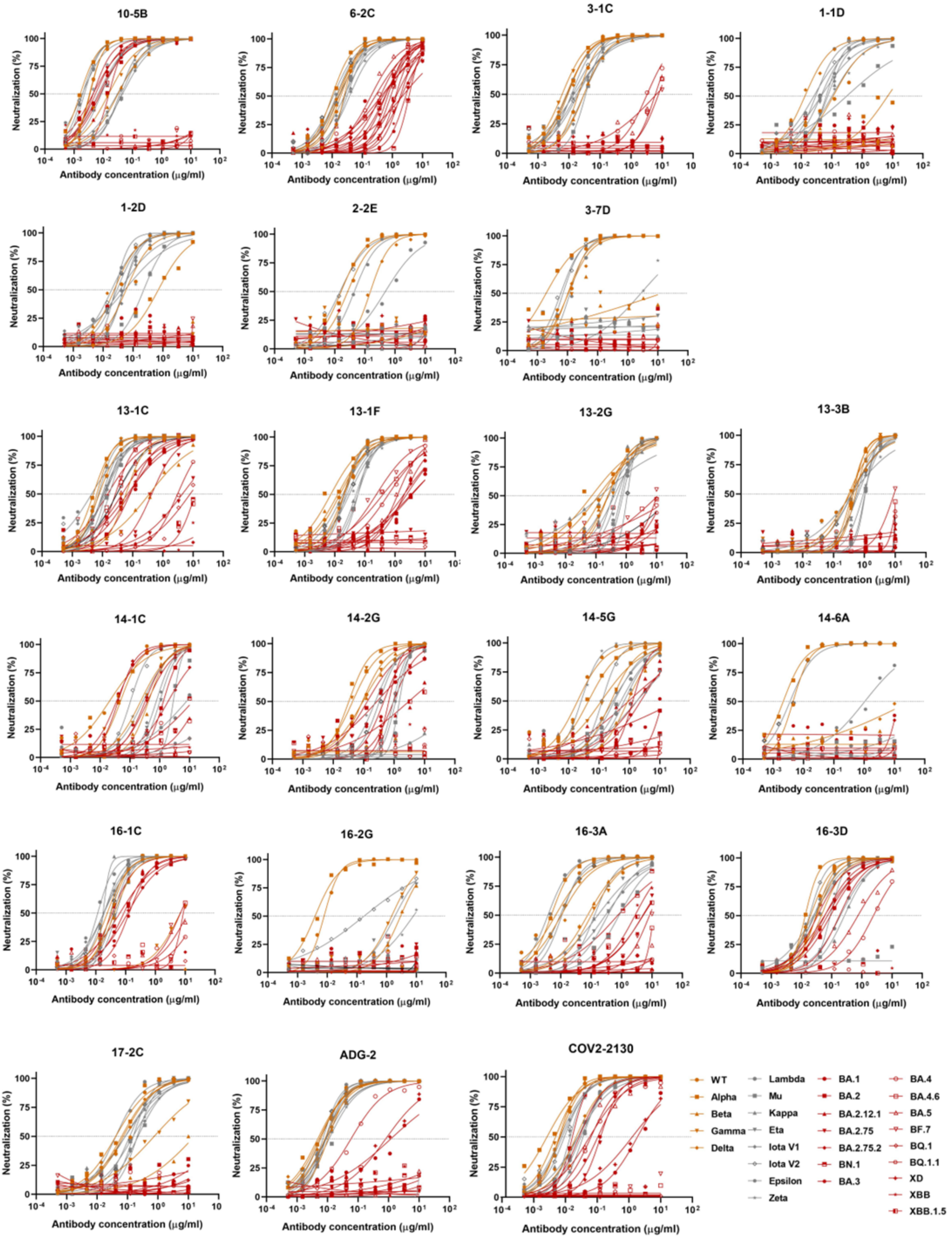
10-5B
6-2C
3-1C
1-1D
1-2D
2-2E
3-7D
13-1C
13-1F
13-2G
13-3B
14-1C
14-2G
14-5G
14-6A
16-1C
16-2G
16-3A
16-3D
17-2C
ADG-2
COV2-2130
Neutralization (%)
Antibody concentration (μg/ml)
WT
Alpha
Beta
Gamma
Delta
Lambda
Mu
Kappa
Eta
Iota V1
Iota V2
Epsilon
Zeta
BA.1
BA.2
BA.2.12.1
BA.2.75
BA.2.75.2
BN.1
BA.3
BA.4
BA.4.6
BA.5
BF.7
BQ.1
BQ.1.1
XD
XBB
XBB.1.5

Supplementary Fig. 5 Antibody neutralization analyzed with pseudovirus SARS-CoV-2 wild type and variants. Pseudovirus neutralization assays were performed on human immunodeficiency virus-1 (HIV-1) pseudotyped with the S protein of SARS-CoV-2 wild type and variants using HeLa-hACE2 target cells. The curves were fitted by nonlinear regression (log [inhibitor] vs. normalized response, variable slope). The dashed line indicates a 50% reduction in viral infectivity. Data for each mAb were obtained from one neutralization experiment of duplicate, represented as mean ± s.d.. Individual mAbs were tested independently with similar results.

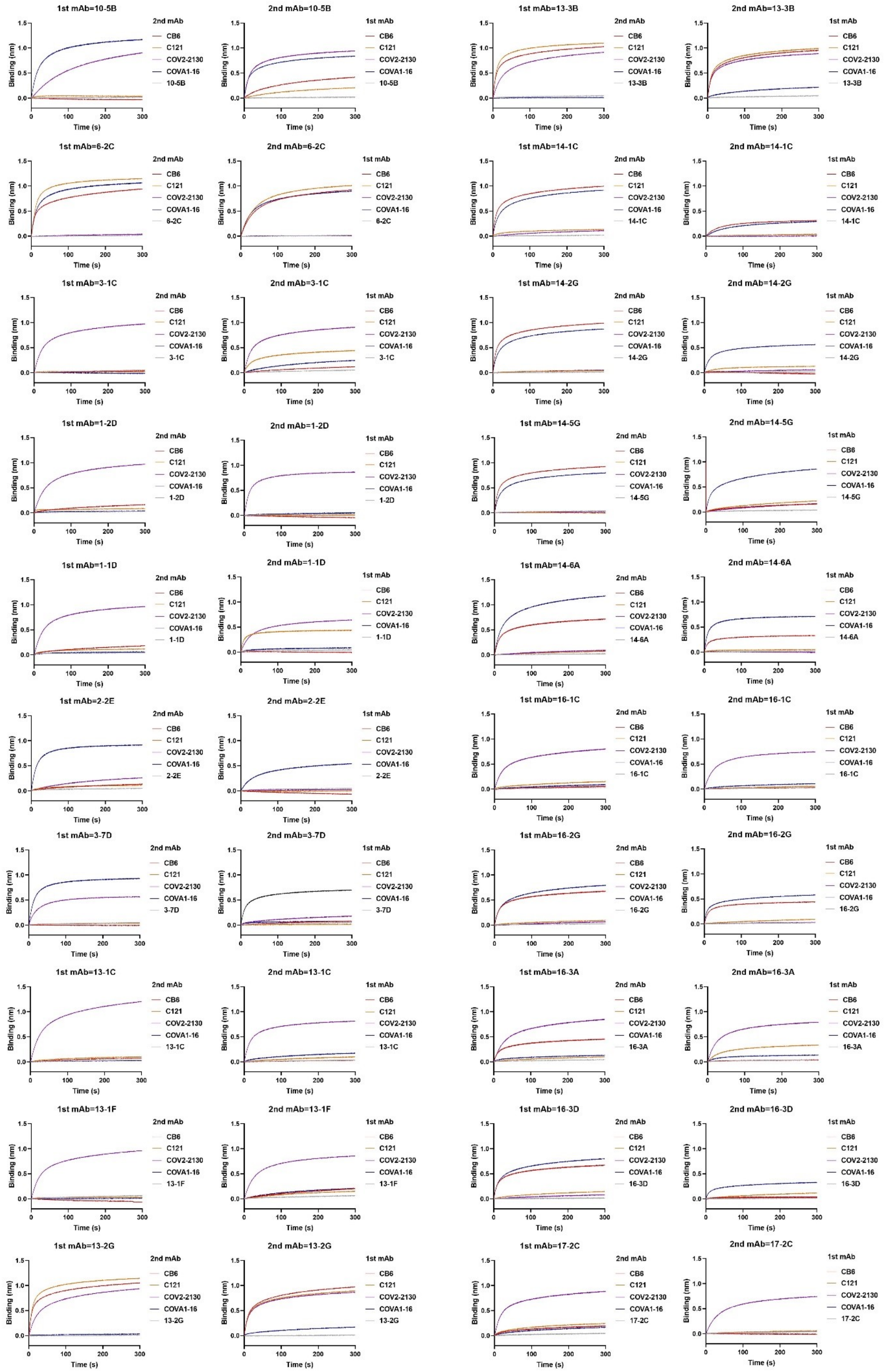
Binding (nm)
Time (s)
1st mAb=10-5B
2nd mAb: CB6, C121, COV2-2130, COVA1-16, 10-5B
2nd mAb=10-5B
1st mAb: CB6, C121, COV2-2130, COVA1-16, 10-5B
1st mAb=13-3B
2nd mAb: CB6, C121, COV2-2130, COVA1-16, 13-3B
2nd mAb=13-3B
1st mAb: CB6, C121, COV2-2130, COVA1-16, 13-3B
1st mAb=6-2C
2nd mAb: CB6, C121, COV2-2130, COVA1-16, 6-2C
2nd mAb=6-2C
1st mAb: CB6, C121, COV2-2130, COVA1-16, 6-2C
1st mAb=14-1C
2nd mAb: CB6, C121, COV2-2130, COVA1-16, 14-1C
2nd mAb=14-1C
1st mAb: CB6, C121, COV2-2130, COVA1-16, 14-1C
1st mAb=3-1C
2nd mAb: CB6, C121, COV2-2130, COVA1-16, 3-1C
2nd mAb=3-1C
1st mAb: CB6, C121, COV2-2130, COVA1-16, 3-1C
1st mAb=14-2G
2nd mAb: CB6, C121, COV2-2130, COVA1-16, 14-2G
2nd mAb=14-2G
1st mAb: CB6, C121, COV2-2130, COVA1-16, 14-2G
1st mAb=1-2D
2nd mAb: CB6, C121, COV2-2130, COVA1-16, 1-2D
2nd mAb=1-2D
1st mAb: CB6, C121, COV2-2130, COVA1-16, 1-2D
1st mAb=14-5G
2nd mAb: CB6, C121, COV2-2130, COVA1-16, 14-5G
2nd mAb=14-5G
1st mAb: CB6, C121, COV2-2130, COVA1-16, 14-5G
1st mAb=1-1D
2nd mAb: CB6, C121, COV2-2130, COVA1-16, 1-1D
2nd mAb=1-1D
1st mAb: CB6, C121, COV2-2130, COVA1-16, 1-1D
1st mAb=14-6A
2nd mAb: CB6, C121, COV2-2130, COVA1-16, 14-6A
2nd mAb=14-6A
1st mAb: CB6, C121, COV2-2130, COVA1-16, 14-6A
1st mAb=2-2E
2nd mAb: CB6, C121, COV2-2130, COVA1-16, 2-2E
2nd mAb=2-2E
1st mAb: CB6, C121, COV2-2130, COVA1-16, 2-2E
1st mAb=16-1C
2nd mAb: CB6, C121, COV2-2130, COVA1-16, 16-1C
2nd mAb=16-1C
1st mAb: CB6, C121, COV2-2130, COVA1-16, 16-1C
1st mAb=3-7D
2nd mAb: CB6, C121, COV2-2130, COVA1-16, 3-7D
2nd mAb=3-7D
1st mAb: CB6, C121, COV2-2130, COVA1-16, 3-7D
1st mAb=16-2G
2nd mAb: CB6, C121, COV2-2130, COVA1-16, 16-2G
2nd mAb=16-2G
1st mAb: CB6, C121, COV2-2130, COVA1-16, 16-2G
1st mAb=13-1C
2nd mAb: CB6, C121, COV2-2130, COVA1-16, 13-1C
2nd mAb=13-1C
1st mAb: CB6, C121, COV2-2130, COVA1-16, 13-1C
1st mAb=16-3A
2nd mAb: CB6, C121, COV2-2130, COVA1-16, 16-3A
2nd mAb=16-3A
1st mAb: CB6, C121, COV2-2130, COVA1-16, 16-3A
1st mAb=13-1F
2nd mAb: CB6, C121, COV2-2130, COVA1-16, 13-1F
2nd mAb=13-1F
1st mAb: CB6, C121, COV2-2130, COVA1-16, 13-1F
1st mAb=16-3D
2nd mAb: CB6, C121, COV2-2130, COVA1-16, 16-3D
2nd mAb=16-3D
1st mAb: CB6, C121, COV2-2130, COVA1-16, 16-3D
1st mAb=13-2G
2nd mAb: CB6, C121, COV2-2130, COVA1-16, 13-2G
2nd mAb=13-2G
1st mAb: CB6, C121, COV2-2130, COVA1-16, 13-2G
1st mAb=17-2C
2nd mAb: CB6, C121, COV2-2130, COVA1-16, 17-2C
2nd mAb=17-2C
1st mAb: CB6, C121, COV2-2130, COVA1-16, 17-2C

Supplementary Fig. 6 Epitope mapping through competitive binding measured by biolayer interferometry (BLI). Twenty neutralizing antibodies were assayed for epitope specificity with 4 structurally defined monoclonal antibodies using wild-type RBD proteins as the capture antigens. CB6 was classified into Class 1, C121 (Class 2), COV2-2130 (Class 3), and COVA1-16 (Class 4). The traces show binding patterns of the second antibody (2nd Ab, or Ab2) to preformed first antibody (1st Ab, or Ab1)–RBD complexes in an in-tandem binning assay.

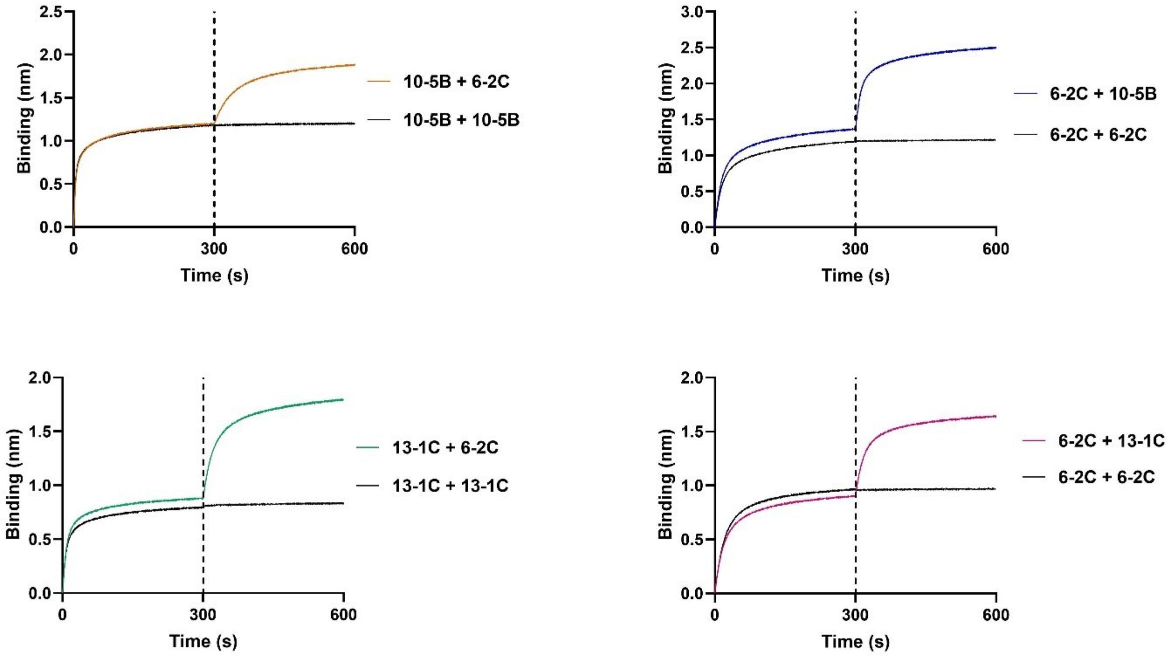

Supplementary Fig. 7 Competitive binding of the monoclonal antibodies 6-2C with 10-5B and 13-1C measured by BLI. Biotinylated antigen (SARS-CoV-2 wild-type RBD) was immobilized on streptavidin sensors and then saturated with the first antibody (1st Ab, or Ab1), followed by the second antibody (2nd Ab, or Ab2). The traces show binding patterns of the second antibody (2nd Ab, or Ab2) to preformed first antibody (1st Ab, or Ab1)–RBD complexes in an in-tandem binning assay.

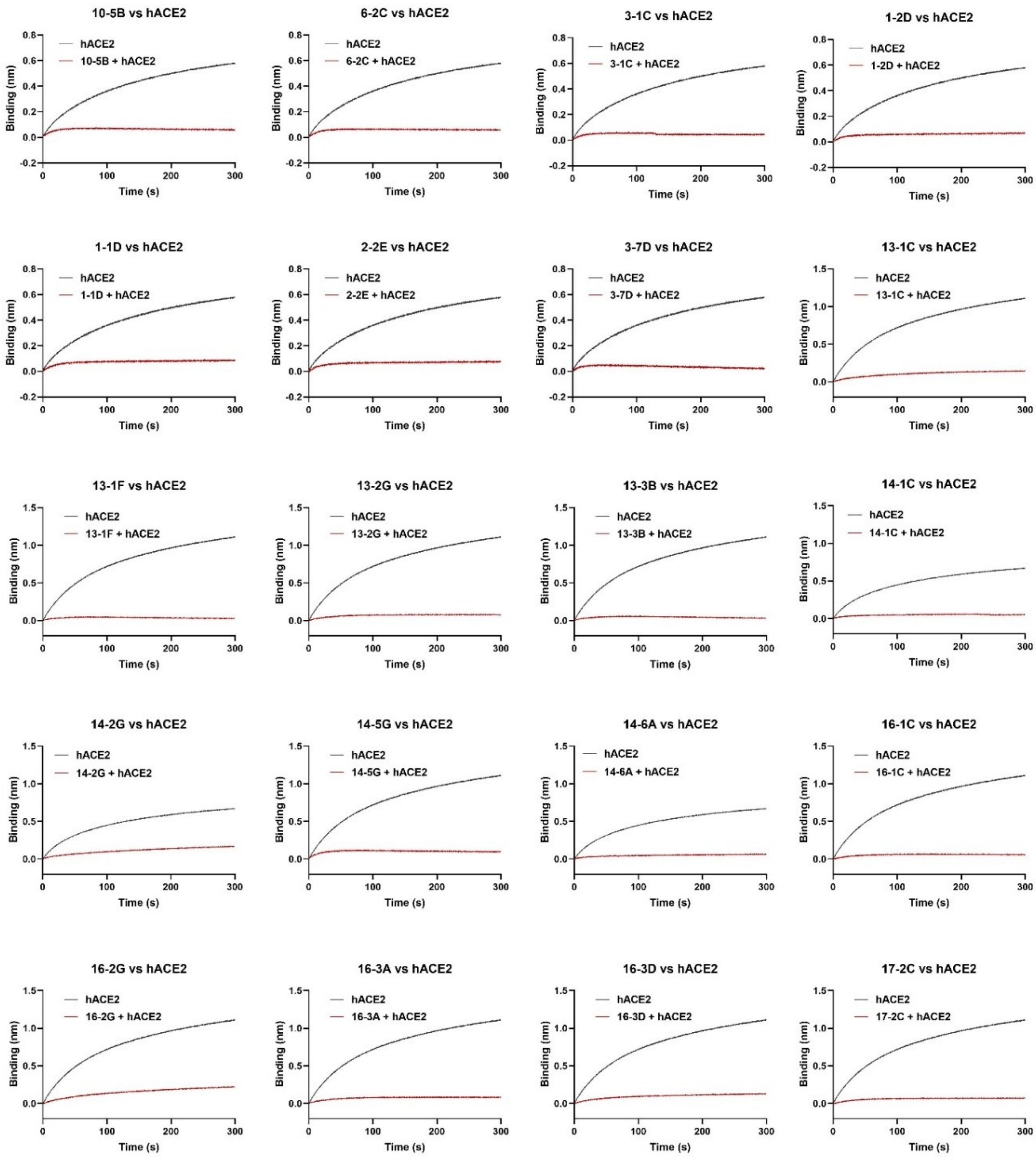

Supplementary Fig. 8 Antibody and hACE2 competition for binding to the SARS-CoV-2 RBD determined by BLI. The traces depict the binding of the hACE2 to preformed antibody–RBD complexes. The level of reduction in the shift of Ab + hACE2 compared to that of the hACE2-only control indicates the blocking capacity of the antibody.

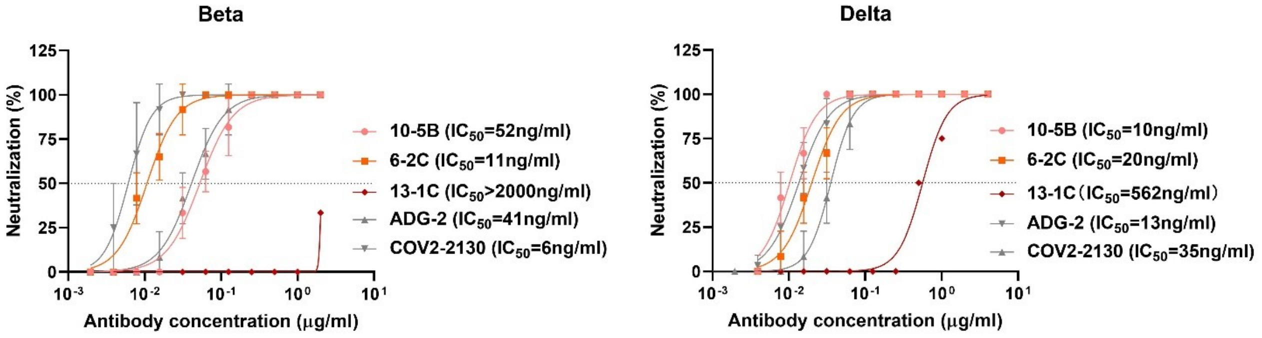

Supplementary Fig. 9 Antibody neutralization analyzed with authentic SARS-CoV-2 Beta and Delta variants. Authentic SARS-CoV-2 neutralization was performed using a cytopathic effect (CPE) assay. The curves were fitted by nonlinear regression (log [inhibitor] vs. normalized response, variable slope). The dashed line indicates a 50% reduction in viral infectivity. Data for each mAb were obtained from one neutralization experiment. Mean ± s.d. of triplicates is shown, except for 13-1C (mean of duplicates). The experiments were independently performed twice with similar results.

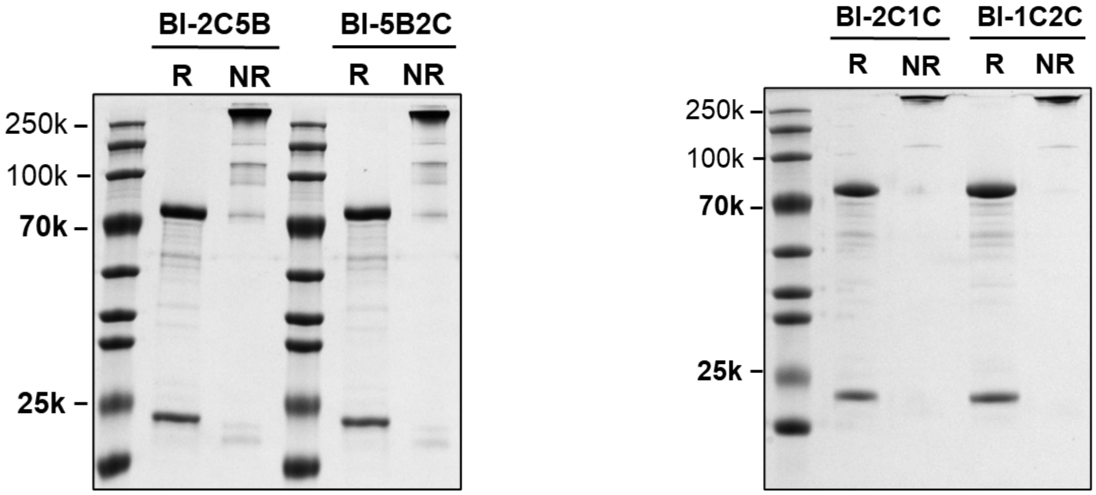

Supplementary Fig. 10 Representative SDS-PAGE analysis of the four bispecific antibodies BI-2C5B, BI-5B2C, BI-2C1C, and BI-1C2C. The molecular masses of the heavy and light chains of are approximately 80 kDa and 25 kDa, respectively. The experiment was performed twice with similar results. R, reduced; NR, non-reduced.

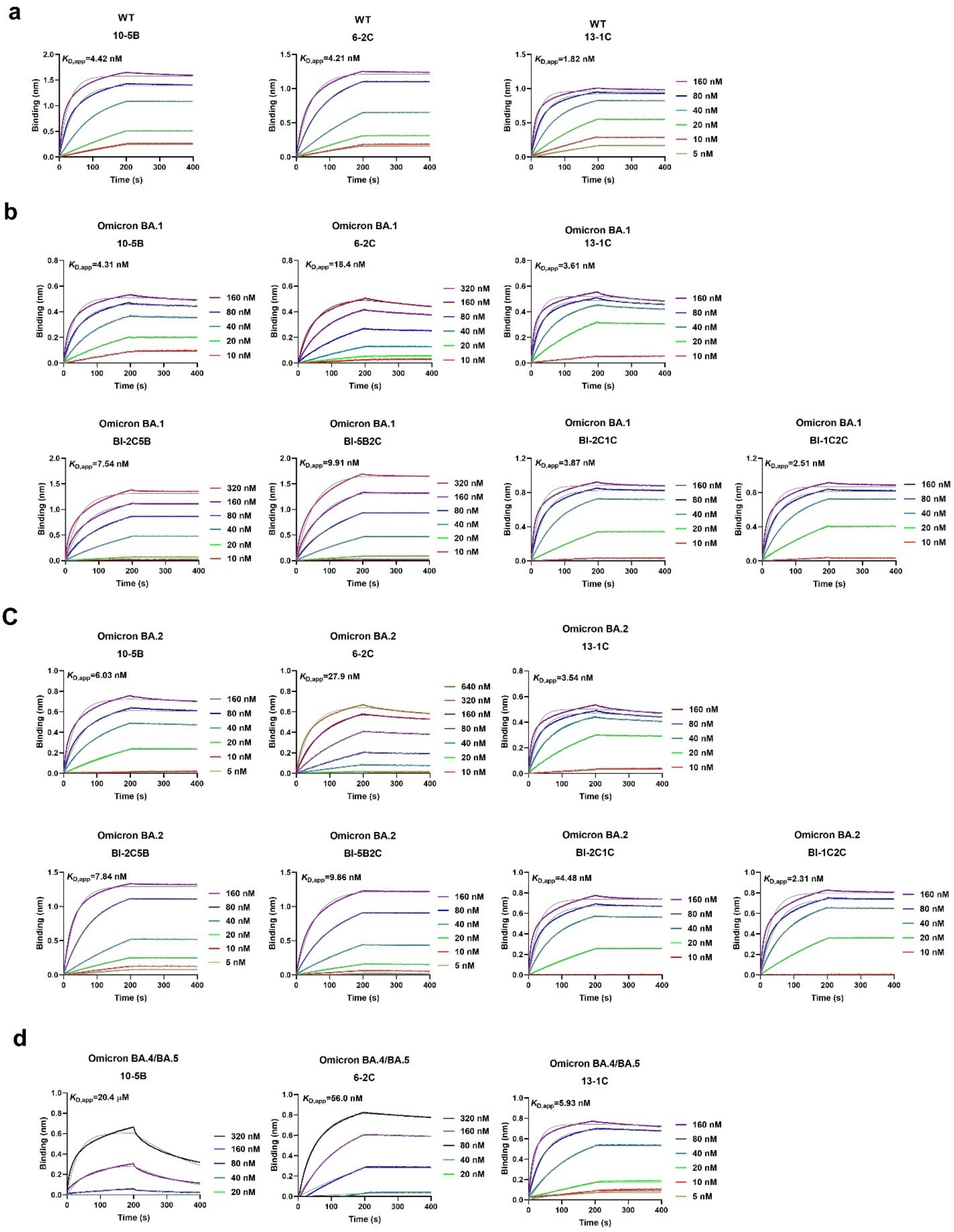

Supplementary Fig. 11 Binding analysis of monoclonal antibodies with SARS-CoV-2 RBDs measured by BLI. Biotinylated RBD of SARS-CoV-2 wild type (WT) (**a**), Omicron BA.1 (**b**), Omicron BA.2 (**c**), and Omicron BA.4/BA.5 (**d**) were loaded onto the surface of SA

biosensors. Individual antibodies were tested at a series of concentrations. The association and dissociation of response curves of the monoclonal antibodies are shown. The grey lines represent fitted curves based on the experimental data. Equilibrium apparent dissociation constants ($K_{D,app}$) are shown above each plot.

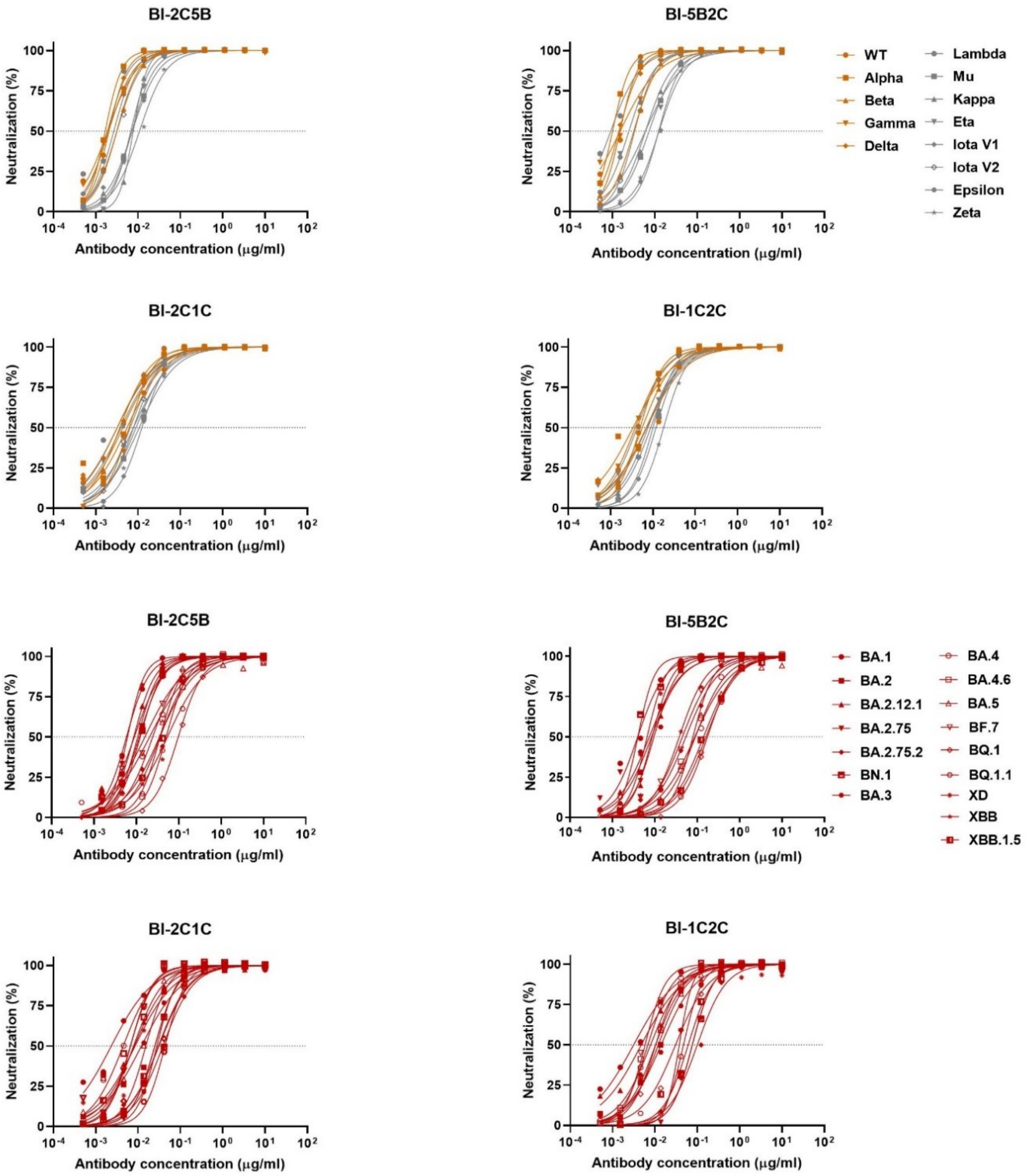

Supplementary Fig. 12 Bispecific antibody neutralization analyzed with SARS-CoV-2 wild-type and variant pseudoviruses. Pseudovirus neutralization assays were performed on human immunodeficiency virus-1 (HIV-1) pseudotyped with the S protein of SARS-CoV-2 wild type and variants using HeLa-hACE2 target cells. The curves were fitted by nonlinear regression (log [inhibitor] vs. normalized response, variable slope). The dashed line indicates a 50% reduction in viral infectivity. Data for each mAb were obtained from one neutralization experiment of duplicate, represented as mean ± s.d.. Individual mAbs were tested independently

with similar results.

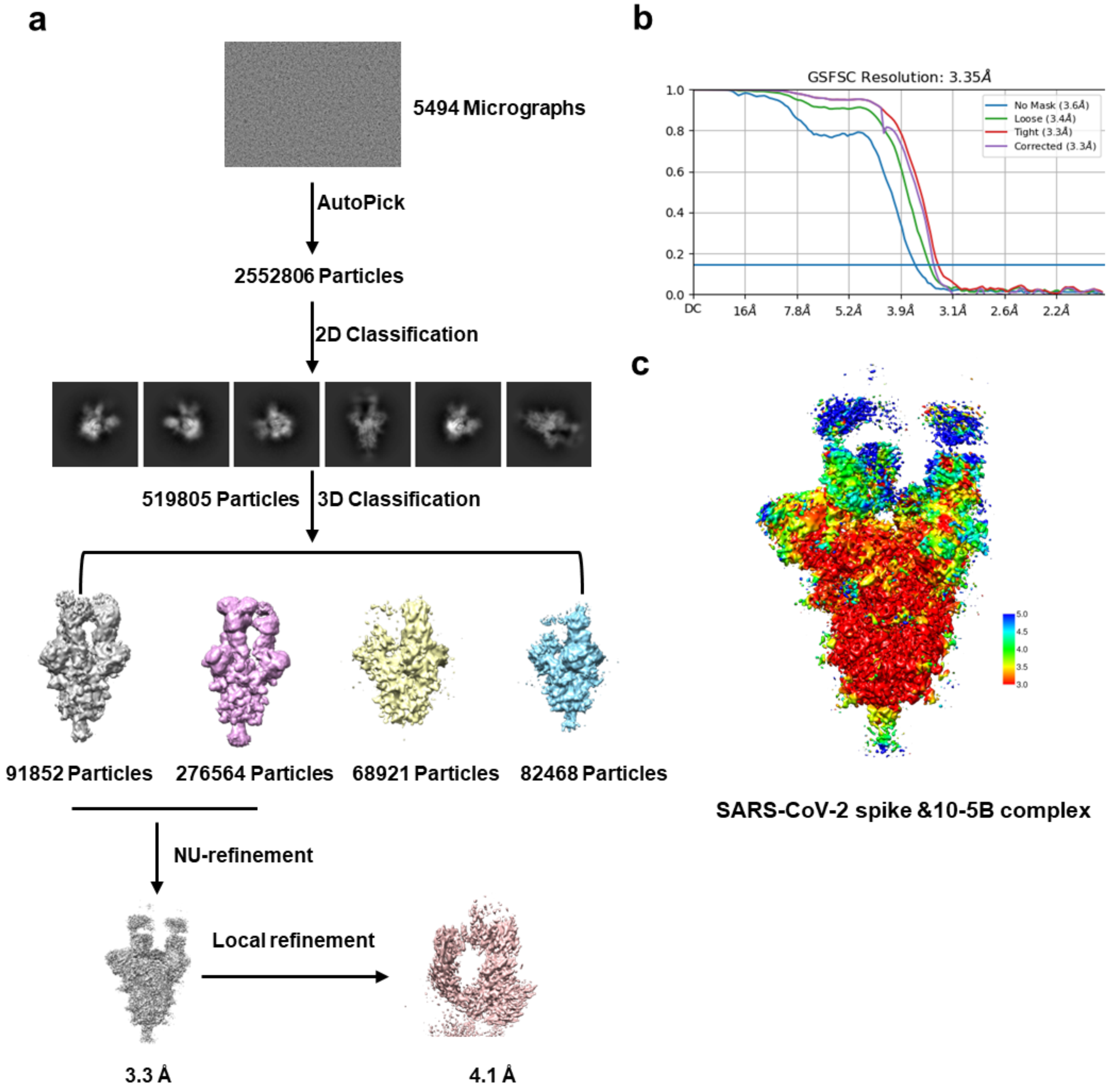

Supplementary Fig. 13 Cryo-EM data processing for wild-type S trimer in complex with 10-5B.

(**a**) Flowcharts for data processing.

(**b**) The gold standard FSC curves of wild-type Spike & 10-5B overall map.

(**c**) Local resolution assessments of wild-type Spike & 10-5B overall map.

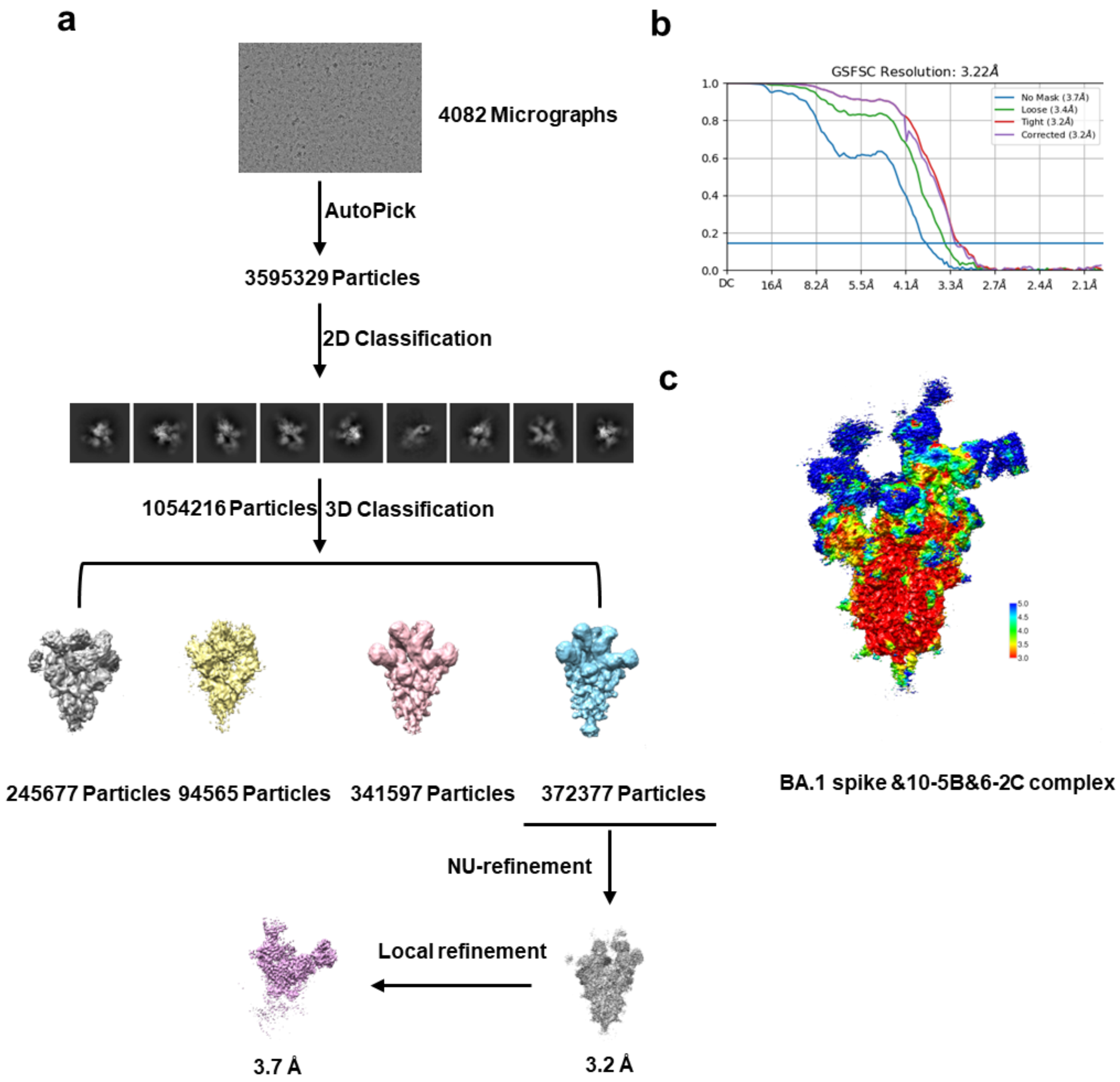

Supplementary Fig. 14 Cryo-EM data processing for Omicron BA.1 S trimer in complex with 10-5B and 6-2C.

(**a**) Flowcharts for data processing.

(**b**) The gold standard FSC curves of Omicron BA.1 Spike & 10-5B & 6-2C overall map.

(**c**) Local resolution assessments of Omicron BA.1 Spike & 10-5B & 6-2C overall map.

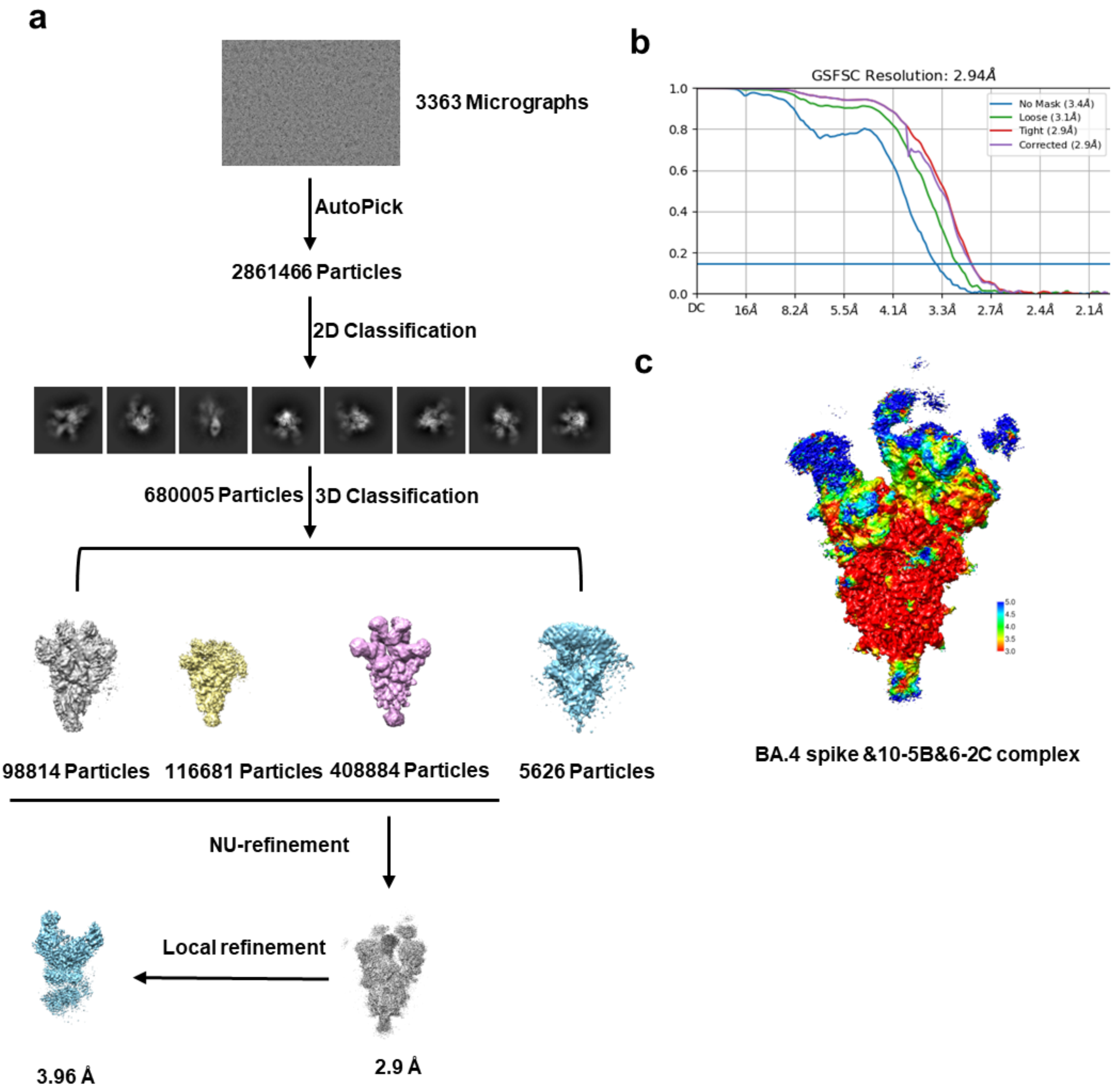

Supplementary Fig. 15 Cryo-EM data processing for Omicron BA.4 S trimer in complex with 10-5B and 6-2C.

(**a**) Flowcharts for data processing.

(**b**) The gold standard FSC curves of Omicron BA.4 Spike & 10-5B & 6-2C overall map.

(**c**) Local resolution assessments of Omicron BA.4 Spike & 10-5B & 6-2C overall map.

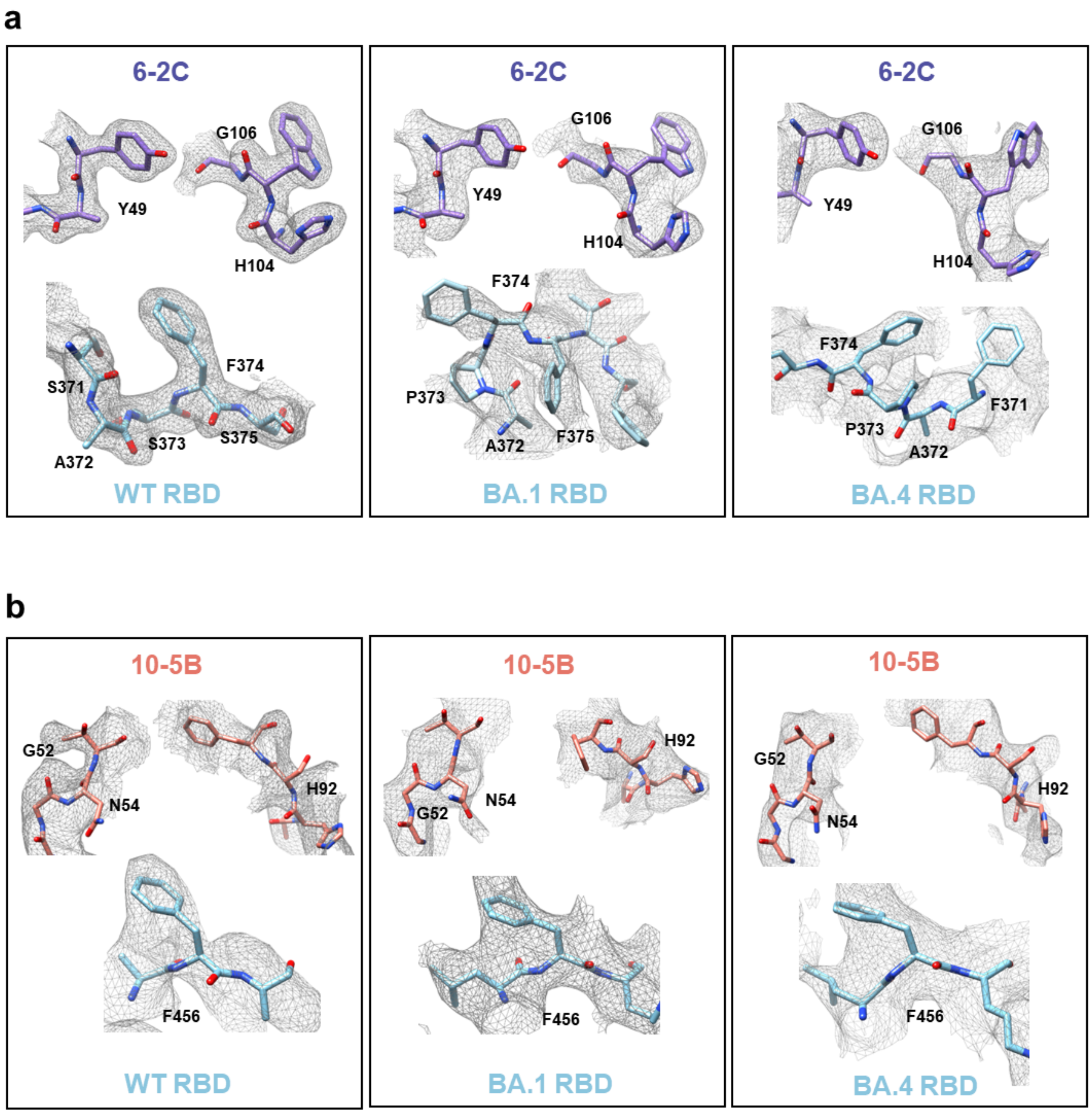

Supplementary Fig. 16 Cryo-EM density maps of the antibody-RBD interface.

(**a**) Cryo-EM density maps of the interface formed between 6-2C and RBD (WT, BA.1, and BA.4), respectively.

(**b**) Cryo-EM density maps of the interface formed between 10-5B and RBD (WT, BA.1, and BA.4), respectively.

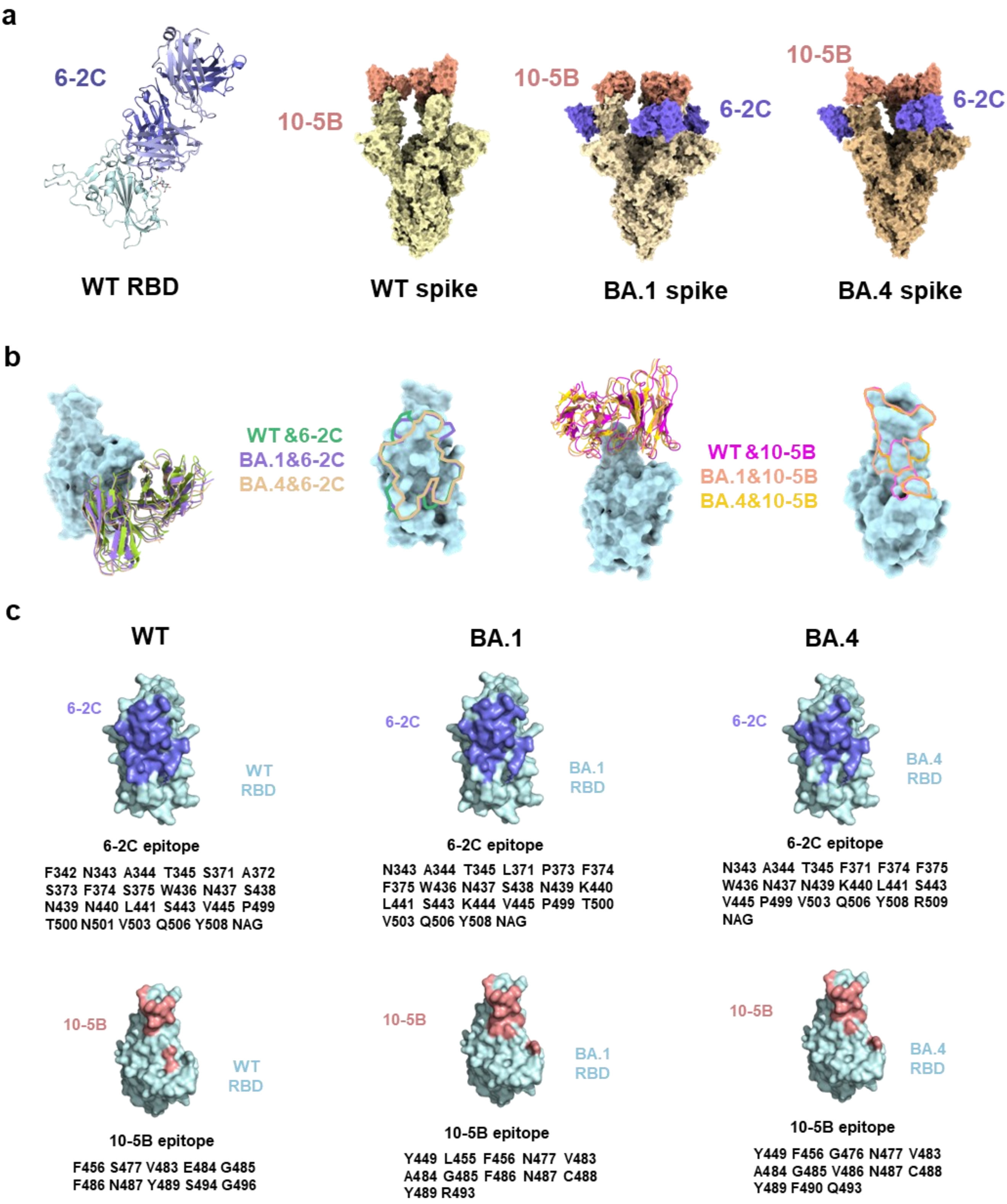

Supplementary Fig. 17 Structural characterization of mAbs 6-2C and 10-5B.

(**a**) Cartoon diagram of one 6-2C Fab (purple) bound to one SARS-CoV-2 RBD (cyan) and cryo-EM structures of WT spike bound with 10-5B Fab (3.3 Å), BA.1 spike bound with 10-5B and 6-2C Fabs (3.2 Å), BA.4 spike bound with 10-5B and 6-2C (2.9 Å) Fabs.

(**b**) The footprints of 6-2C and 10-5B on WT RBD, BA.1 RBD, and BA.4 RBD, respectively.

(**c**) The epitope residues of 6-2C and 10-5B on WT RBD, BA.1 RBD, and BA.4 RBD, respectively.

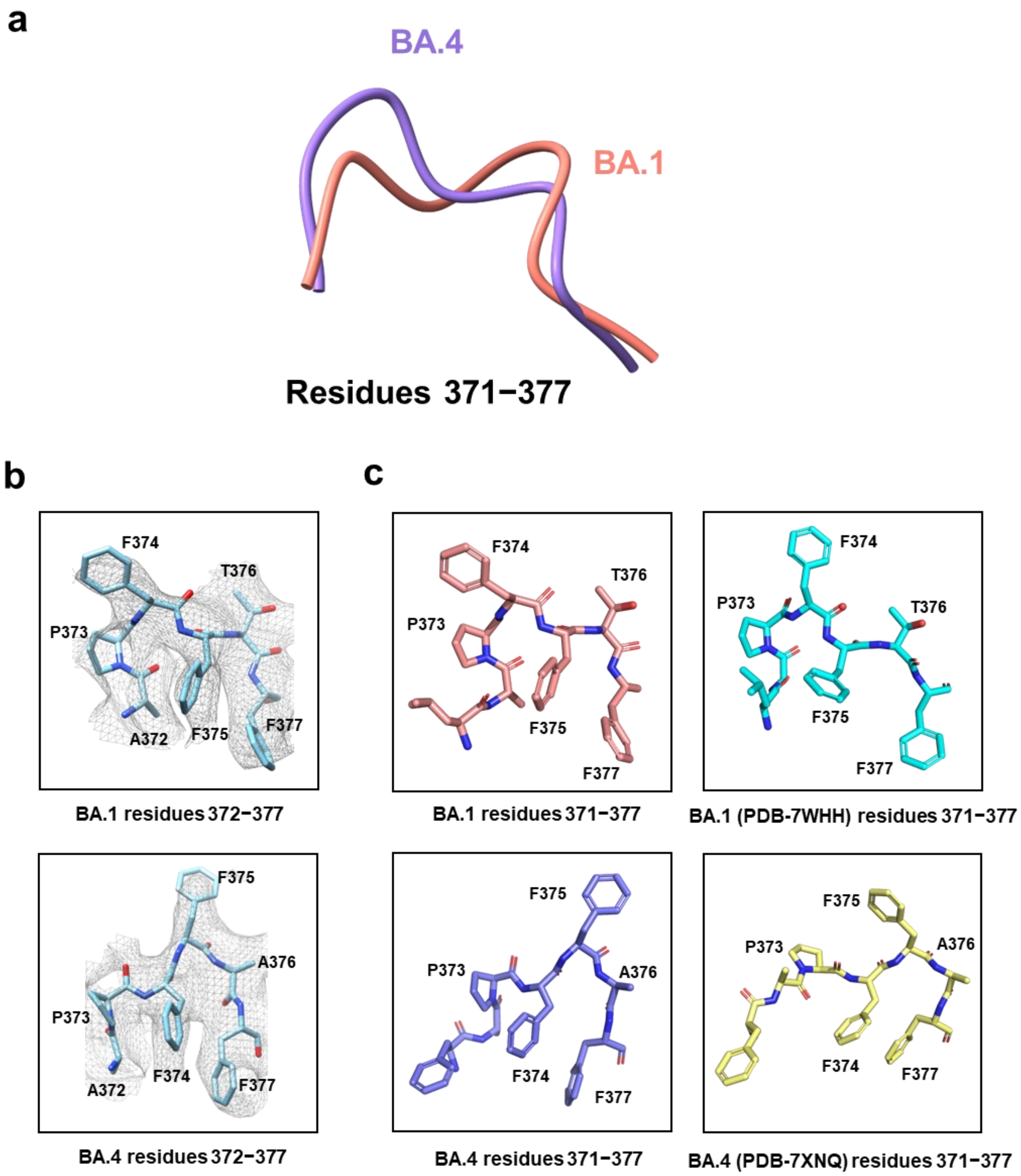

Supplementary Fig. 18 The loop with residues 371–377 in the spike of Omicron BA.1 and BA.4.

(**a**) Cartoon diagram of 371−377 loop in BA.1 spike (salmon) superposed with that of BA.4 spike (purple).

(**b**) Density map of BA.1 and BA.4 residues 372−377.

(**c**) Side-chain conformation of residues 371−377 in BA.1 spike (salmon, this work; cyan, 7WHH) and BA.4 spike (purple, this work; yellow, 7XNQ).

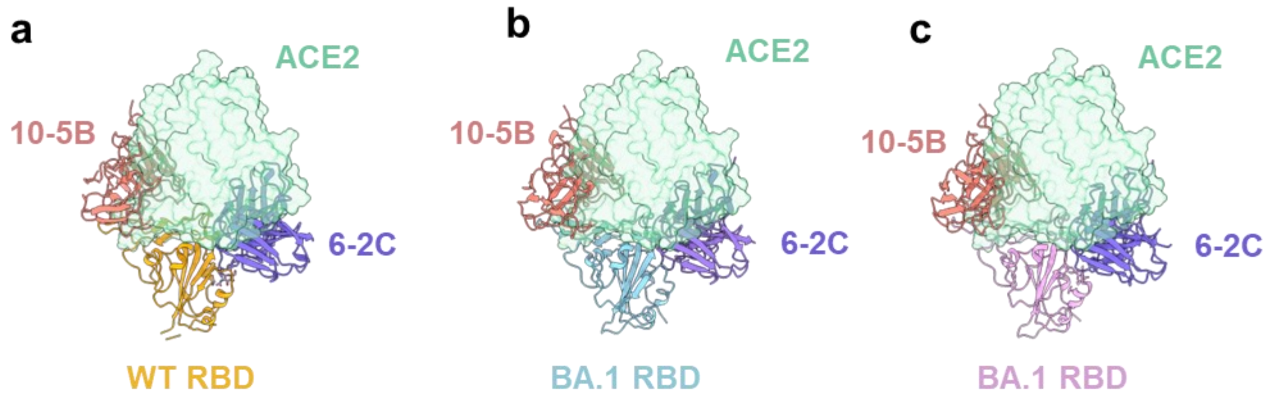

Supplementary Fig. 19 Superimposition of 10-5B-bound, 6-2C-bound, and ACE2-bound [PDB: 6M0J] SARS-CoV-2 RBD structures. View of 10-5B (salmon), 6-2C (purple) and ACE2 (green) footprints on WT RBD (yellow) (**a**), BA.1 RBD (cyan) (**b**), and BA.4 RBD (pink) (**c**).

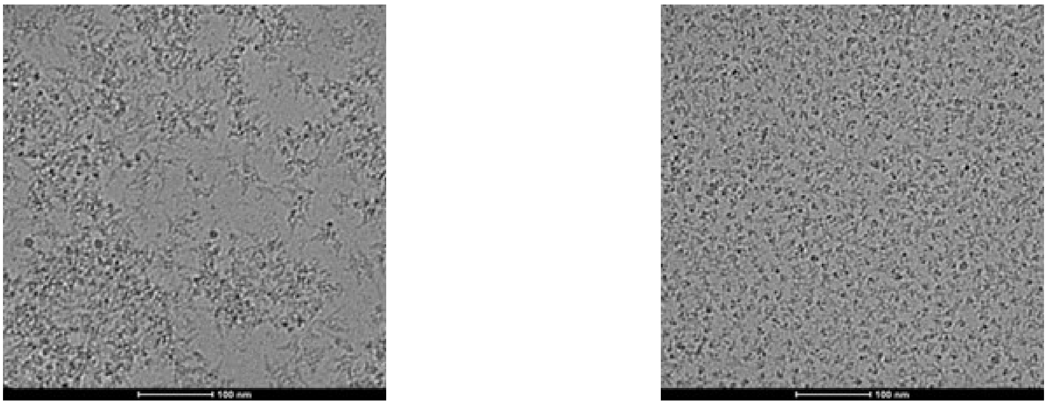

BA.4 & BI-2C5B

BA.4 & 10-5B Fab & 6-2C Fab

Supplementary Fig. 20 Structural characterization of the bsAb BI-2C5B. Left: Representative cryo-EM image of BA.4 spike and BI-2C5B complex. Right: Representative cryo-EM images of BA.4 spike, 10-5B Fab, and 6-2C Fab ternary complex. Scale bar, 100 nm. The experiment was performed twice with similar results.

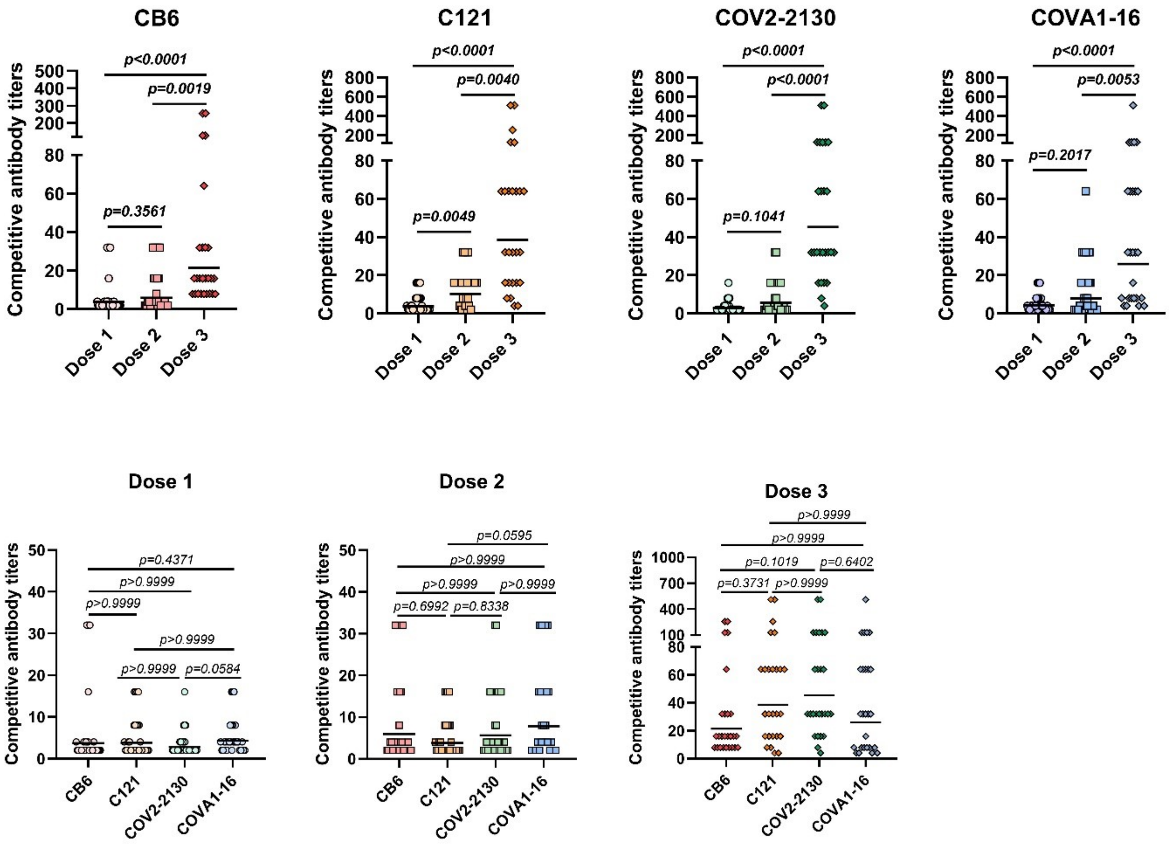

Supplementary Fig. 21 Plasma antibodies specific for different epitopes on SARS-CoV-2 RBD measured by competitive ELISA. Plasma collected one month after the first, second, and third vaccine doses were assayed for competitive titers with 4 structurally defined monoclonal antibodies using wild-type RBD protein as the coated antigen. CB6 was classified into Class 1, C121 (Class 2), COV2-2130 (Class 3), and COVA1-16 (Class 4). Statistical significance was determined by two-sided Kruskal–Wallis test with subsequent Dunn's multiple comparisons.

Supplementary Table 1 Characteristics of the participants

ID	Age range at enrollment (years)	Sex	Race	Vaccine platform received	1st to 2nd dose (days)	2nd to 3rd dose (days)	1st visit to 1st dose (days)	1st dose to 2nd visit (days)	1st dose to 3rd visit (days)	2nd dose to 4th visit (days)	2nd dose to 5th visit (days)	2nd dose to 6th visit (days)	2nd dose to 7th visit (days)	3rd dose to 8th visit (days)
1	20-29	M	Asian	BBIBP-CorV	28	314	0	15	26	14	28	85	168	29
2	20-29	M	Asian	BBIBP-CorV	28	207	0	15	26	14	28	85	168	109
3	20-29	M	Asian	BBIBP-CorV	28	315	0	15	26	14	28	85	168	28
4	20-29	F	Asian	BBIBP-CorV	28	278	0	15	26	14	28	85	168	38
5	20-29	M	Asian	BBIBP-CorV	28	242	0	15	26	14	28	85	168	74
6	20-29	F	Asian	BBIBP-CorV	28	242	0	15	26	14	28	85	168	74
7	20-29	M	Asian	BBIBP-CorV	28	242	0	15	26	14	28	85	168	74
8	20-29	M	Asian	BBIBP-CorV	28	285	0	15	26	14	28	85	168	31
9	30-39	F	Asian	BBIBP-CorV	28	316	0	15	26	14	28	85	168	27
10	20-29	M	Asian	BBIBP-CorV	28	279	0	15	26	14	28	85	168	37
11	30-39	M	Asian	BBIBP-CorV	28	280	0	15	26	14	28	85	168	36
12	20-29	M	Asian	BBIBP-CorV	28	242	0	15	26	14	28	85	168	74
13	30-39	M	Asian	BBIBP-CorV	28	231	0	15	26	14	28	85	168	85
14	40-49	M	Asian	BBIBP-CorV	28	203	0	15	26	14	28	85	168	113
15	40-49	F	Asian	BBIBP-CorV	28	230	0	15	26	14	28	85	-	-
16	30-39	F	Asian	BBIBP-CorV	28	287	0	15	26	14	28	85	168	32
17	20-29	F	Asian	BBIBP-CorV	28	292	0	15	26	14	28	85	168	24
18	20-29	M	Asian	BBIBP-CorV	28	287	0	15	26	14	28	85	168	29
19	20-29	F	Asian	BBIBP-CorV	28	318	0	15	26	14	28	85	168	25
20	50-59	F	Asian	BBIBP-CorV	28	280	0	15	26	14	28	85	168	36
21	30-39	M	Asian	BBIBP-CorV	28	288	0	15	26	14	28	85	168	28
22	30-39	M	Asian	BBIBP-CorV	28	-	0	15	26	14	28	85	168	-
23	30-39	M	Asian	BBIBP-CorV	28	290	0	15	26	14	28	85	168	26
24	50-59	M	Asian	BBIBP-CorV	28	280	0	15	26	14	28	85	168	36
25	30-39	F	Asian	BBIBP-CorV	28	282	0	15	26	14	28	85	168	34
26	20-29	M	Asian	BBIBP-CorV	28	282	0	15	26	14	28	85	168	34
27	20-29	M	Asian	BBIBP-CorV	28	281	0	15	26	14	28	85	168	35
28	30-39	F	Asian	BBIBP-CorV	28	286	0	15	26	14	28	85	168	30

M, male; F, female.

Supplementary Table 2 SARS-CoV-2 variants tested in this study

Variants	Nonsynonymous mutations present in SARS-CoV-2 spike
**Alpha**	H69-, V70-, 144 Y-, N501Y, A570D, D614G, P681H, T761I, S982A, D1118H
**Beta**	L18F, D80A, D215G, L242-, A243-, L244-, K417N, E484K, N501Y, D614G, A701V
**Gamma**	L18F, T20N, P26S, D138Y, R190S, K417T, E484K, N501Y, D614G, H655Y, T1027I
**Delta**	T19R, G142D, E156-, F157-, R158G, L452R, T478K, D614G, P681R, D950N
**Kappa**	T95I, G142D, E154K, L452R, E484Q, D614G, P681R, Q1071H
**Lambda**	G75V, T76I, R246N, S247-, Y248-, L249-, T250-, P251-, G252-, D253-, L452Q, F490S, D614G, T859N
**Mu**	T95I, Y144T, Y145S, ins146N, R346K, E484K, N501Y, D614G, P681H, D950N
**Eta**	Q52R, A67V, H69-, V70-, Y144-, E484K, D614G, Q677H, F888L
**Iota v1**	T95I, D253G, E484K, D614G, A701V
**Iota v2**	T95I, D253G, S477N, D614G, Q957R
**Epsilon**	S13I, W152C, L452R, D614G
**Zeta**	E484K, D614G, V1176F
**BA.1**	A67V, H69-, V70-, T95I, G142D, V143-, Y144-, Y145-, N211-, L212I, ins214EPE, G339D, S371L, S373P, S375F, K417N, N440K, G446S, S477N, T478K, E484A, Q493R, G496S, Q498R, N501Y, Y505H, T547K, D614G, H655Y, N679K, P681H, N764K, D796Y, N856K, Q954H, N969K, L981F
**BA.2**	T19I, L24-, P25-, P26-, A27S, G142D, V213G, G339D, S371F, S373P, S375F, T376A, D405N, R408S, K417N, N440K, S477N, T478K, E484A, Q493R, Q498R, N501Y, Y505H, D614G, H655Y, N679K, P681H, N764K, D796Y, Q954H, N969K
**BA.2.12.1**	T19I, L24-, P25-, P26-, A27S, G142D, V213G, G339D, S371F, S373P, S375F, T376A, D405N, R408S, K417N, N440K, L452Q, S477N, T478K, E484A, Q493R, Q498R, N501Y, Y505H, D614G, H655Y, N679K, P681H, S704L, N764K, D796Y, Q954H, N969K
**BA.2.75**	T19I, L24-, P25-, P26-, A27S, G142D, K147E, W152R, F157L, I210V, V213G, G257S, G339H, S371F, S373P, S375F, T376A, D405N, R408S, K417N, N440K, G446S, N460K, S477N, T478K, E484A, Q498R, N501Y, Y505H, D614G, H655Y, N679K, P681H, N764K, D796Y, Q954H, N969K
**BA.2.75.2**	T19I, L24-, P25-, P26-, A27S, G142D, K147E, W152R, F157L, I210V, V213G, G257S, G339H, R346T, S371F, S373P, S375F, T376A, D405N, R408S, K417N, N440K, G446S, N460K, S477N, T478K, E484A, F486S, Q498R, N501Y, Y505H, D614G, H655Y, N679K, P681H, N764K, D796Y, Q954H, N969K
**BN.1**	T19I, L24-, P25-, P26-, A27S, G142D, K147E, W152R, F157L, I210V, V213G, G257S, G339H, R346T, K356T, S371F, S373P, S375F, T376A, D405N, R408S, K417N, N440K, G446S, N460K, S477N, T478K, E484A, F490S, Q498R, N501Y, Y505H, D614G, H655Y, N679K, P681H, N764K, D796Y, Q954H, N969K
**BA.3**	A67V, H69-, V70-, T95I, G142D, V143-, Y144-, Y145-, N211-, L212I, G339D, S371F, S373P, S375F, D405N, K417N, N440K, G446S, S477N, T478K, E484A, Q493R, Q498R, N501Y, Y505H, D614G, H655Y, N679K, P681H, N764K, D796Y, Q954H, N969K

**BA.4**	T19I, L24-, P25-, P26-, A27S, H69-, V70-, G142D, V213G, G339D, S371F, S373P, S375F, T376A, D405N, R408S, K417N, N440K, L452R, S477N, T478K, E484A, F486V, Q498R, N501Y, Y505H, D614G, H655Y, N658S, N679K, P681H, N764K, D796Y, Q954H, N969K
**BA.4.6**	T19I, L24-, P25-, P26-, A27S, H69-, V70-, G142D, V213G, G339D, R346T, S371F, S373P, S375F, T376A, D405N, R408S, K417N, N440K, L452R, S477N, T478K, E484A, F486V, Q498R, N501Y, Y505H, D614G, H655Y, N658S, N679K, P681H, N764K, D796Y, Q954H, N969K
**BA.5**	T19I, L24-, P25-, P26-, A27S, H69-, V70-, G142D, V213G, G339D, S371F, S373P, S375F, T376A, D405N, R408S, K417N, N440K, L452R, S477N, T478K, E484A, F486V, Q498R, N501Y, Y505H, D614G, H655Y, N679K, P681H, N764K, D796Y, Q954H, N969K
**BF.7**	T19I, L24-, P25-, P26-, A27S, H69-, V70-, G142D, V213G, G339D, R346T, S371F, S373P, S375F, T376A, D405N, R408S, K417N, N440K, L452R, S477N, T478K, E484A, F486V, Q498R, N501Y, Y505H, D614G, H655Y, N679K, P681H, N764K, D796Y, Q954H, N969K
**BQ.1**	T19I, L24S, P25-, P26-, A27-, H69-, V70-, G142D, V213G, G339D, S371F, S373P, S375F, T376A, D405N, R408S, K417N, N440K, K444T, L452R, N460K, S477N, T478K, E484A, F486V, Q498R, N501Y, Y505H, D614G, H655Y, N679K, P681H, N764K, D796Y, Q954H, N969K
**BQ.1.1**	T19I, L24S, P25-, P26-, A27-, H69-, V70-, G142D, V213G, G339D, R346T, S371F, S373P, S375F, T376A, D405N, R408S, K417N, N440K, K444T, L452R, N460K, S477N, T478K, E484A, F486V, Q498R, N501Y, Y505H, D614G, H655Y, N679K, P681H, N764K, D796Y, Q954H, N969K
**XD**	T19R, A27S, T95I, G142D,156-,157-, R158G, N211-, L212I, ins214EPE, G339D, S371L, S373P, S375F, K417N, N440K, G446S, S477N, T478K, E484A, Q493R, G496S, Q498R, N501Y, Y505H, T547K, D614G, H655Y, N679K, P681H, N764K, D796Y, N856K, Q954H, N969K, L981F
**XBB**	T19I, L24-, P25-, P26-, A27S, V83A, G142D, Y145Q, H146-, Q183E, V213E, G339H, R346T, L368I, S371F, S373P, S375F, T376A, D405N, R408S, K417N, N440K, V445P, G446S, N460K, S477N, T478K, E484A, F486S, F490S, Q498R, N501Y, Y505H, D614G, H655Y, N679K, P681H, N764K, D796Y, Q954H, N969K
**XBB.1.5**	T19I, L24-, P25-, P26-, A27S, V83A, G142D, Y145Q, H146-, Q183E, V213E, G252V, G339H, R346T, L368I, S371F, S373P, S375F, T376A, D405N, R408S, K417N, N440K, V445P, G446S, N460K, S477N, T478K, E484A, F486P, F490S, Q498R, N501Y, Y505H, D614G, H655Y, N679K, P681H, N764K, D796Y, Q954H, N969K

Supplementary Table 3 Characteristics of subjects selected for antibody isolation

Subject ID	mAbs obtained at	ELISA Binding (titer)		Pseudovirus neutralization (ID_{50})			
		Anti-Spike IgG	Anti-RBD IgG	WT	Previous VOCs (GMTs)	Previous VOIs (GMTs)	Omicron subvariants (GMTs)
1	Dose 2 + 1 M	8100	8100	101	40	25	7
	Dose 2 + 3 M	2700	2700	21	n.d.	n.d.	n.d.
	Dose 2 + 6 M	900	300	5	n.d.	n.d.	n.d.
	Dose 3 + 1 M	24300	8100	641	196	188	30
3	Dose 2 + 1 M	8100	8100	86	60	78	7
	Dose 2 + 3 M	2700	900	11	n.d.	n.d.	n.d.
	Dose 2 + 6 M	2700	900	5	n.d.	n.d.	n.d.
	Dose 3 + 1 M	8100	8100	223	98	115	19
4	Dose 2 + 1 M	8100	8100	255	72	98	5
	Dose 2 + 3 M	8100	2700	64	n.d.	n.d.	n.d.
	Dose 2 + 6 M	8100	900	63	n.d.	n.d.	n.d.
	Dose 3+ 1 M	24300	24300	896	440	525	25
11	Dose 2 + 1 M	900	900	22	67	45	8
	Dose 2 + 3 M	900	300	5	n.d.	n.d.	n.d.
	Dose 2 + 6 M	300	300	5	n.d.	n.d.	n.d.
	Dose 3 + 1 M	24300	8100	255	108	175	40
17	Dose 2 + 1 M	8100	8100	109	48	44	5
	Dose 2 + 3 M	8100	2700	24	n.d.	n.d.	n.d.
	Dose 2 + 6 M	2700	900	37	n.d.	n.d.	n.d.
	Dose 3 + 1 M	8100	8100	483	188	152	20
25	Dose 2 + 1 M	8100	2700	248	41	29	7
	Dose 2 + 3 M	2700	900	55	n.d.	n.d.	n.d.
	Dose 2 + 6 M	900	900	42	n.d.	n.d.	n.d.
	Dose 3 + 1 M	8100	8100	644	196	100	18

M, month; W, week. GMTs, geometric mean titers; n.d., not determined.

Supplementary Table 4 Characteristics of neutralizing antibodies

mAb	Subject ID	Obtained at	Heavy chain					Light chain			
			IGHV	IGHD	IGHJ	CDR3 length	SHM (%)	IGK(L)V	IGK(L)J	CDR3 length	SHM (%)
**6-2C**	3	Dose 2 + 3 M	3-30*04, 3-30-3*03	6-19*01	4*02	15	5.1	KV1-16*01	KJ4*01	9	3.6
**10-5B**	17	Dose 2 + 6 M	3-53*01	6-13*01, 6-19*01, 6-25*01	3*02	13	6.2	KV1-12*01	KJ2*01, KJ2*02, KJ3*01	9	3.5
**3-1C**	1	Dose 2 + 3 M	3-53*01	2-2*01, 2-2*03, 3-22*01	4*02	12	3.4	KV1-39*01, V1D-39*01	KJ4*01	11	2.5
**1-1D**	1	Dose 2 + 1 M	3-53*01	1-7*01	6*02	13	1.0	LV1-51*01	LJ3*02	11	2.0
**1-2D**	1	Dose 2 + 1 M	5-10-1*01	6-13*01	5*02	20	0.7	KV1-39*01, KV1D-39*01	KJ1*01	9	1.4
**2-2E**	1	Dose 2 + 1 M	3-53*01	3-22*01	4*02	16	0	LV4-69*01	LJ3*02	10	0.3
**3-7D**	4	Dose 2 + 3 M	3-66*02	3-22*01	4*02	18	2.1	KV4-1*01	KJ1*01	10	0.7
**13-1C**	11	Dose 3 + 1 M	3-66*01	4-17*01	4*02	11	4.4	KV3-20*01	KJ1*01	9	4.2
**13-1F**	11	Dose 3 + 1 M	3-53*04	2-15*01, 4-23*01	3*02	11	5.8	KV1-9*01	KJ2*01	11	1.8
**13-2G**	11	Dose 3 + 1 M	4-61*02, 4-61*09	5-18*01, 5-5*01	6*04	12	6.5	LV6-57*03	LJ3*02	9	3.1
**13-3B**	3	Dose 3 + 1 M	3-23*04	2-8*01	4*02	17	4.1	LV6-57*02	LJ3*02	10	0.7
**14-1C**	1	Dose 3 + 1 M	5-10-1*01	2-21*01, 2-21*02	4*02	8	9	LV6-57*01	LJ2*01, LJ3*01	9	4.8
**14-2G**	3	Dose 3 + 1 M	1-69*09	5-12*01	4*02	17	3.7	LV1-40*01	LJ2*01, LJ3*01, LJ3*02	11	1.7
**14-5G**	25	Dose 3 + 1 M	1-46*01, 1-46*02	2-2*01, 2-2*02, 2-2*03	6*02	22	2.7	LV1-40*01	LJ1*01	10	0.3
**14-6A**	25	Dose 3 + 1 M	3-30*04, 3-30-3*03	3-10*01	6*02	21	4.1	LV3-1*01	LJ1*01	10	2.9
**16-1C**	1	Dose 3 + 1 M	3-53*01	4-17*01	4*02	11	3.4	KV3-20*01	KJ3*01	9	2.1
**16-2G**	4	Dose 3 + 1 M	1-69*09	3-22*01	3*02	19	3.1	KV4-*01, KV4-1*02	KJ2*01	9	1.3
**16-3A**	17	Dose 3 + 1 M	3-30*04, 3-30-3*03	3-16*01, 3-16*02	4*02	10	6.1	KV1-12*01, KV1-12*02, KV1D-12*02	KJ2*01	9	4.1
**16-3D**	17	Dose 3 + 1 M	4-61*01	2-2*01, 2-2*02, 2-2*03	3*02	16	5.4	KV1-33*01, KV1D-33*01	KJ2*01	9	1.4
**17-2C**	4	Dose 3 + 1M	3-66*01	1-1*01, 1-20*01, 1-7*01	4*02, 5*02	11	21.9	KV1-12*01, KV1-12*02, KV1D-12*01	KJ5*01	9	0.7

The program IGBLAST was used to analyze germline genes, germline divergence and the degree of somatic hypermutation rate (SHM), the framework region (FR) and the loop length of CDR3 for each antibody clone. The CDR3 length was calculated in terms of amino acids.

Supplementary Table 5 Neutralization profile of monoclonal antibodies against SARS-CoV-2 variants

	Variants	Nonsynonymous mutations present in the RBD	Number of mAbs lost neutralization activity (IC_{50} > 10 µg/ml)			Number of mAbs with fold change in IC_{50} values relative to WT > 3 (including those lost neutralization)		
			Total (n=20)	Dose 2 (n=7)	Dose 3 (n=13)	Total (n=20)	Dose 2 (n=7)	Dose 3 (n=13)
**Previously circulating variants of concern (VOCs)**	Alpha	N501Y	0	0	0	2	2	0
	Beta	K417N, E484K, N501Y	7	4	3	13	5	8
	Gamma	K417T, E484K, N501Y	5	4	1	13	5	8
	Delta	L452R, T478K	4	0	4	6	1	5
**Previously circulating variants of interest (VOIs)**	Lambda	L452Q, F490S	4	0	4	9	1	8
	Mu	R346K, E484K, N501Y	6	2	4	14	4	10
	Kappa	L452R, E484Q	6	2	4	11	2	9
	Eta	E484K	3	2	1	12	3	9
	Iota v1	E484K	4	2	2	10	3	7
	Iota v2	S477N	0	0	0	3	0	3
	Epsilon	L452R	3	0	3	5	0	5
	Zeta	E484K	2	1	1	10	3	7
**Omicron subvariants**	BA.1	G339D, S371L, S373P, S375F, K417N, N440K, G446S, S477N, T478K, E484A, Q493R, G496S, Q498R, N501Y, Y505H	11	5	6	16	6	10
	BA.2	G339D, S371F, S373P, S375F, T376A, D405N, R408S, K417N, N440K, S477N, T478K, E484A, Q493R, Q498R, N501Y, Y505H	12	5	7	18	7	11
	BA.2.12.1	G339D, S371F, S373P, S375F, T376A, D405N, R408S, K417N, N440K, L452Q, S477N, T478K, E484A, Q493R, Q498R, N501Y, Y505H	12	5	7	18	6	12
	BA.2.75	G339H, S371F, S373P, S375F, T376A, D405N, R408S, K417N, N440K, G446S, N460K, S477N, T478K, E484A, Q498R, N501Y, Y505H	12	5	7	18	6	12
	BA.2.75.2	G339H, R346T, S371F, S373P, S375F, T376A, D405N, R408S, K417N, N440K, G446S, N460K, S477N, T478K, E484A, F486S, Q498R, N501Y, Y505H	17	6	11	20	7	13
	BN.1	G339H, R346T, K356T, S371F, S373P, S375F, T376A, D405N, R408S, K417N, N440K, G446S,	17	5	12	20	7	13

		N460K, S477N, T478K, E484A, F490S, Q498R, N501Y, Y505H						
	BA.3	G339D, S371F, S373P, S375F, D405N, K417N, N440K, G446S, S477N, T478K, E484A, Q493R, Q498R, N501Y, Y505H	12	5	7	18	6	12
	BA.4/BA.5	G339D, S371F, S373P, S375F, T376A, D405N, R408S, K417N, N440K, L452R, S477N, T478K, E484A, F486V, Q498R, N501Y, Y505H	15	5	10	20	7	13
	BA.4.6	G339D, R346T, S371F, S373P, S375F, T376A, D405N, R408S, K417N, N440K, L452R, S477N, T478K, E484A, F486V, Q498R, N501Y, Y505H	15	5	10	20	7	13
	BF.7	G339D, R346T, S371F, S373P, S375F, T376A, D405N, R408S, K417N, N440K, L452R, S477N, T478K, E484A, F486V, Q498R, N501Y, Y505H	13	5	8	19	7	12
	BQ.1	G339D, S371F, S373P, S375F, T376A, D405N, R408S, K417N, N440K, K444T, L452R, N460K, S477N, T478K, E484A, F486V, Q498R, N501Y, Y505H	18	6	12	20	7	13
	BQ.1.1	G339D, R346T, S371F, S373P, S375F, T376A, D405N, R408S, K417N, N440K, K444T, L452R, N460K, S477N, T478K, E484A, F486V, Q498R, N501Y, Y505H	18	6	12	20	7	13
	XD	G339D, S371L, S373P, S375F, K417N, N440K, G446S, S477N, T478K, E484A, Q493R, G496S, Q498R, N501Y, Y505H	11	5	6	17	6	11
	XBB	G339H, R346T, L368I, S371F, S373P, S375F, T376A, D405N, R408S, K417N, N440K, V445P, G446S, N460K, S477N, T478K, E484A, F486S, F490S, Q498R, N501Y, Y505H	18	6	12	20	7	13
	XBB.1.5	G339H, R346T, L368I, S371F, S373P, S375F, T376A, D405N, R408S, K417N, N440K, V445P, G446S, N460K, S477N, T478K, E484A, F486P, F490S, Q498R, N501Y, Y505H	17	6	11	20	7	13

Supplementary Table 6 Crystallization data collection and refinement statistics

	SARS-CoV-2 RBD&6-2C complex (PDB-7X2H)
**Data collection**	
Space group	P 21 21 2
Unit cell dimensions	
a, b, c (Å)	179.05 72.00 112.76
α, β, γ (°)	90 90 90
Resolution range (Å)	50−2.15 (2.20−2.15)*
Rmerge (%)	16.60 (218.50)*
$I/\sigma I$	16.40 (2.140)*
Completeness (%)	99.14 (93.35)*
Redundancy	12.90 (12.20)*
CC1/2	0.99 (0.61)*
**Refinement**	
Resolution (Å)	31.52−2.10
No. reflections	84721 (7867)*
Rwork/Rfree (%)	23.29/27.19
No. atoms	
Protein	9501
Ligands	28
B-factor (Å^2)	
Protein	46.86
Ligands	47.71
R.m.s deviations	
Bond length (Å)	0.01
Bond angles (°)	1.31
Ramachandran plot	
Favored (%)	95.58
Allowed (%)	4.17
Otliers (%)	0.25

*Values in parentheses are for highest-resolution shell.

Supplementary Table 7 Cryo-EM data collection, refinement, and validation statistics

	SARS-CoV-2 spike & 10-5B complex (PDB-7XD2)	SARS-CoV-2 BA.1 spike & 10-5B&6-2C complex (PDB-8H08)	SARS-CoV-2 BA.4 spike & 10-5B&6-2C complex (PDB-8H07)
**Data collection and processing**			
Magnification	29000	29000	29000
Voltage (kV)	300	300	300
Electron exposure (e–/Å^2)	50	50	50
Defocus range (μm)	-1.3 to -1.5	-1.3 to -1.5	-1.3 to -1.5
Pixel size (Å)	0.97	0.97	0.97
Symmetry imposed	C1	C1	C1
Initial particle images (no.)	2552806	3595329	2861466
Final particle images (no.)	368416	372377	624379
Local map resolution (Å)	3.3	3.2	2.9
FSC threshold	0.143	0.143	0.143
Map resolution range (Å)	3.3−3.4	3.2−3.3	2.9−3.0
**Refinement**			
Initial model used (PDB code)	7DWY	7DWY	7DWY
Model resolution (Å)	3.3	3.2	2.9
FSC threshold	0.143	0.143	0.143
Model resolution range (Å)	3.3−3.4	3.2−3.3	2.9−3.0
Map sharpening B factor (Å^2)	135.7	109.4	113.0
Model composition			
Non-hydrogen atoms	28084	31277	31320
Protein residues	3753	4173	4187
B factors (Å^2)			
Protein	71.37	34.78	51.06
R.m.s. deviations			
Bond lengths (Å)	0.014	0.013	0.013
Bond angles (°)	1.999	2.021	1.929
Validation			
MolProbity score	1.88	1.96	1.97
Clashscore	9.45	9.9	9.77
Poor rotamers (%)	0.91	1.19	1.16
Ramachandran plot			
Favored (%)	94.47	94.35	93.80
Allowed (%)	4.91	5.11	5.54
Disallowed (%)	0.62	0.54	0.66

Supplementary Table 8 Characteristics of subjects with high percentages of Spike-specific memory B cells after the booster dose

Subject ID	Age (years)	Sex	Samples obtained at	ELISA Binding (titer)		Pseudovirus neutralization (ID_{50})			
				Anti-Spike IgG	Anti-RBD IgG	WT	Previous VOCs (GMTs)	Previous VOIs (GMTs)	Omicron subvariants (GMTs)
8	24	M	Dose 2 + 1 M	8100	2700	21	14	8	6
			Dose 2 + 3 M	900	300	5	n.d.	n.d.	n.d.
			Dose 2 + 6 M	900	300	21	n.d.	n.d.	n.d.
			Dose 3 + 1 M	24300	24300	766	192	330	27
11	33	M	Dose 2 + 1 M	2700	2700	22	67	45	8
			Dose 2 + 3 M	900	300	5	n.d.	n.d.	n.d.
			Dose 2 + 6 M	300	300	5	n.d.	n.d.	n.d.
			Dose 3 + 1 M	24300	8100	255	108	175	40
16	33	F	Dose 2 + 1 M	8100	8100	61	29	25	9
			Dose 2 + 3 M	2700	2700	19	n.d.	n.d.	n.d.
			Dose 2 + 6 M	2700	900	10	n.d.	n.d.	n.d.
			Dose 3 + 1 M	72900	72900	3120	643	547	24
27	23	M	Dose 2 + 1 M	8100	2700	18	22	9	6
			Dose 2 + 3 M	2700	900	10	n.d.	n.d.	n.d.
			Dose 2 + 6 M	2700	900	20	n.d.	n.d.	n.d.
			Dose 3 + 1 M	72900	72900	1117	405	255	18

GMTs, geometric mean titers; M, male; F, female; n.d., not determined.
