# Supplementary material for: Author Correction: Inactivated vaccine-elicited potent antibodies can broadly neutralize SARS-CoV-2 circulating variants
Source: Nat Commun. 2026 Oct 30;17:11289. doi: 10.1038/s41467-026-79108-1 (PMC13722683; doi:10.1038/s41467-026-79108-1)
Supplement: Supplementary file 1 [file 41467_2026_79108_MOESM1_ESM.pdf]

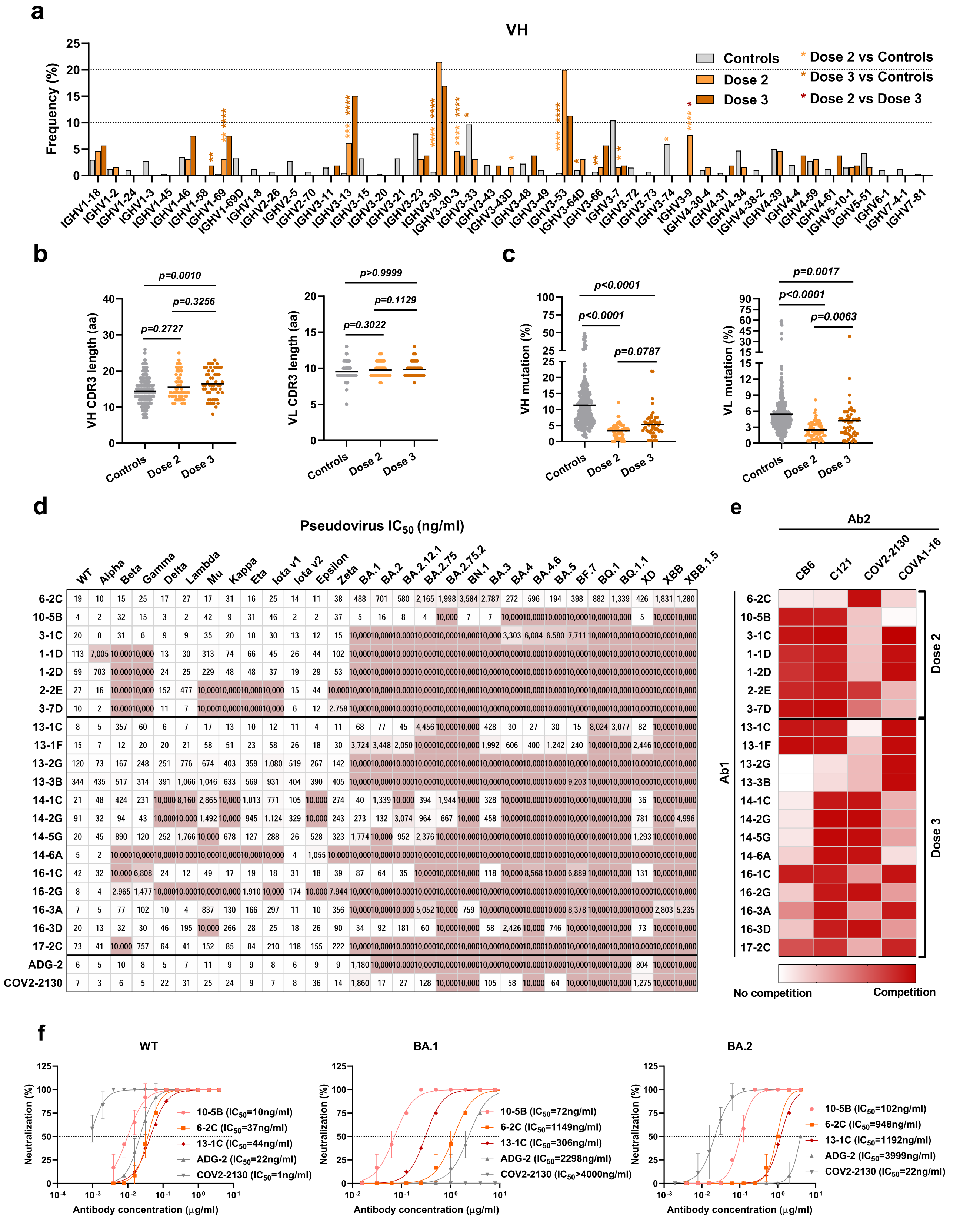

and Supplementary Fig. 12). The bsAbs remained effective against Omicron subvariants that bear escape mutations making them resistant to the individual mAbs (Fig. 3e). To confirm the activity of the bsAbs, we performed a cytopathic effect (CPE) inhibition assay with authentic viruses. All the bsAbs efficiently neutralized WT SARS-CoV-2 (IC_{50} of 2–204 ng/ml), Beta (IC_{50} of 34–220 ng/ml), Delta (IC_{50} of 14–317 ng/ml), Omicron BA.1 (IC_{50} of 3–58 ng/ml) and Omicron BA.2 (IC_{50} of 15–306 ng/ml) (Fig. 3f). Nevertheless, BI-2C5B and BI-5B2C displayed stronger neutralization potency against the live viruses tested than BI-2C1C and BI-1C2C (Fig. 3f). We conclude that the in vitro binding and neutralization properties of the bsAbs make them preferable over their parental antibodies.

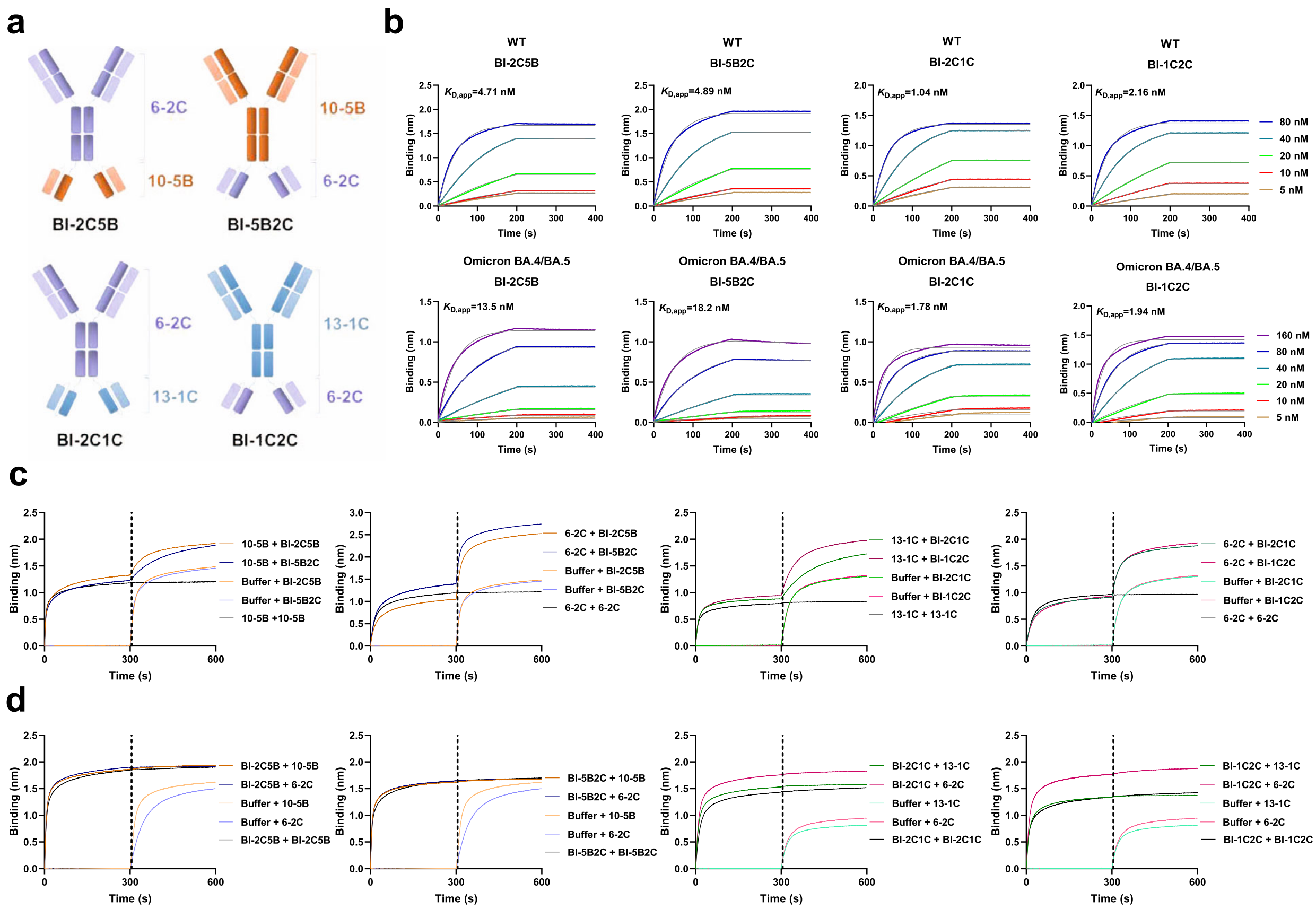

e Pseudovirus IC_{50} (ng/ml)

	WT	Alpha	Beta	Gamma	Delta	Lambda	Mu	Kappa	Eta	Iota v1	Iota v2	Epsilon	Zeta	BA.1	BA.2	BA.2.12.1	BA.2.75	BA.2.75.2	BN.1	BA.3	BA.4	BA.4.6	BA.5	BF.7	BQ.1	BQ.1.1	XD	XBB	XBB.1.5
BI-2C5B	2	2	3	2	2	2	7	8	7	7	3	3	11	6	11	9	9	35	12	12	24	36	20	16	97	54	6	50	36
BI-5B2C	1	1	3	2	2	1	7	6	6	12	3	2	13	4	9	10	7	54	4	8	91	79	88	45	175	162	7	38	142
BI-2C1C	5	4	4	6	3	3	9	8	4	12	7	8	11	2	12	8	28	39	25	15	4	7	9	7	30	43	8	25	31
BI-1C2C	7	3	6	4	5	4	9	7	7	12	10	8	18	3	12	5	38	100	77	14	7	8	10	6	31	47	10	77	61

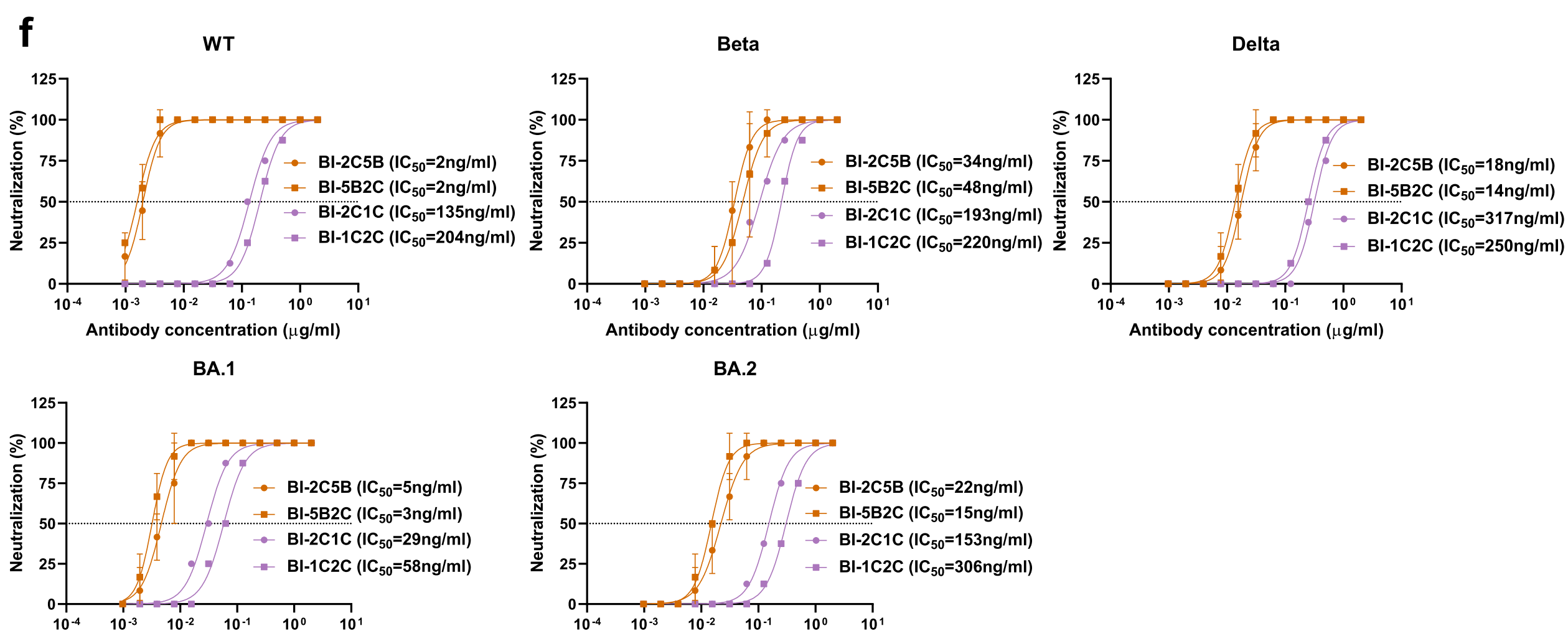

relatively decreased binding activity of 6-2C against the Omicron variant. Furthermore, the epitope of 6-2C overlapped or clashed with the ACE2-binding site on the SARS-CoV-2 RBD, indicating competition with ACE2, as supported by the BLI assay (Fig. 5c and Supplementary Fig. 19), suggesting that 6-2C neutralized SARS-CoV-2 through the inhibition of virus/host cell interactions.

We have shown that 10-5B is highly potent against the pseudoviruses of WT SARS-CoV-2 and nearly all tested VOCs and VOIs, including Omicron BA.1, BA.2, BA.2.12.1, BA.3 and XD subvariants, with IC_{50} values in the range of 2–46 ng/ml (Fig. 2d). However, its potency was significantly decreased to more than 10,000 ng/ml against Omicron BA.4/5 and its subvariants (Fig. 2d). Similar to the analysis of the

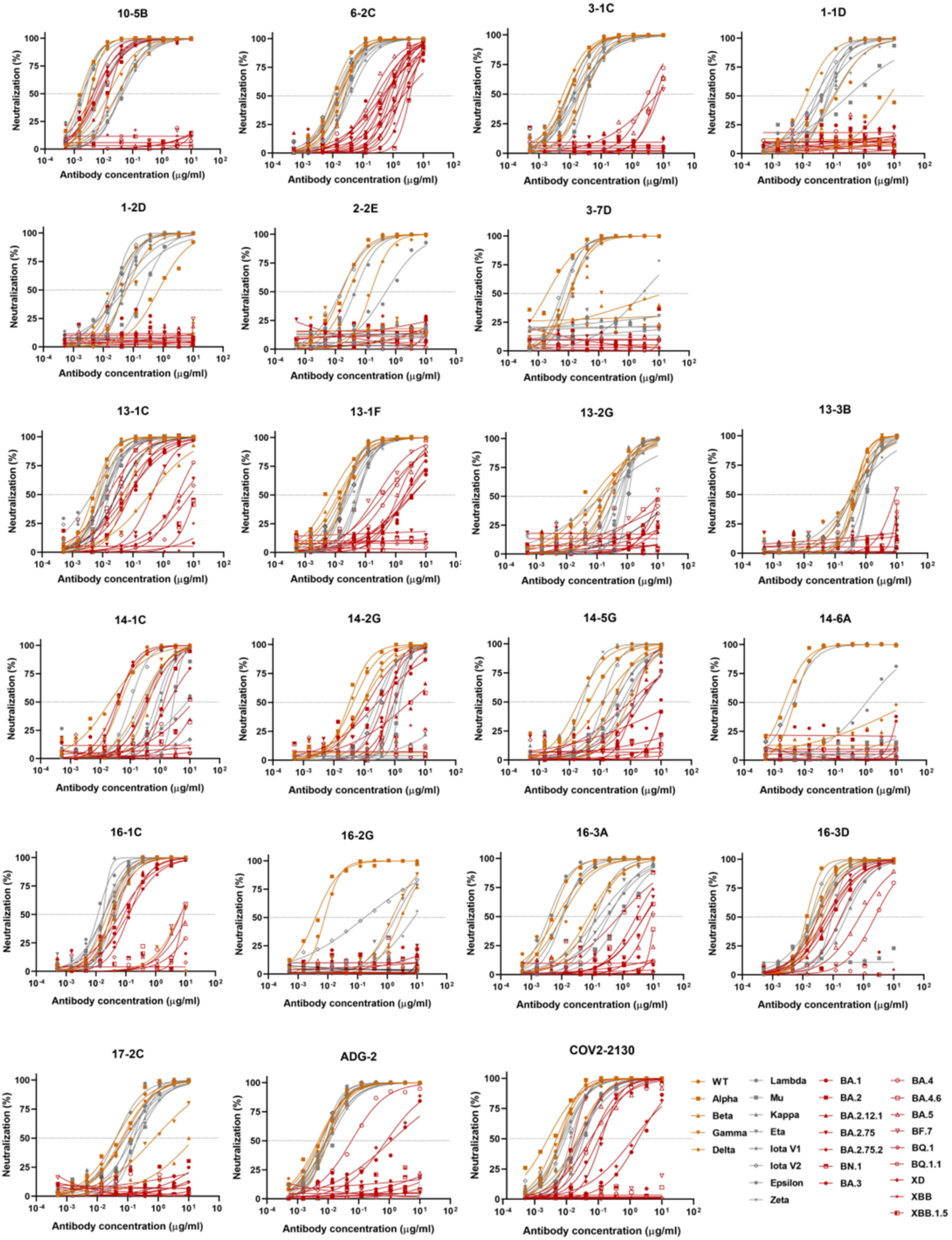
10-5B
6-2C
3-1C
1-1D
1-2D
2-2E
3-7D
13-1C
13-1F
13-2G
13-3B
14-1C
14-2G
14-5G
14-6A
16-1C
16-2G
16-3A
16-3D
17-2C
ADG-2
COV2-2130
Neutralization (%)
Antibody concentration (μg/ml)
WT
Alpha
Beta
Gamma
Delta
Lambda
Mu
Kappa
Eta
Iota V1
Iota V2
Epsilon
Zeta
BA.1
BA.2
BA.2.12.1
BA.2.75
BA.2.75.2
BN.1
BA.3
BA.4
BA.4.6
BA.5
BF.7
BQ.1
BQ.1.1
XD
XBB
XBB.1.5

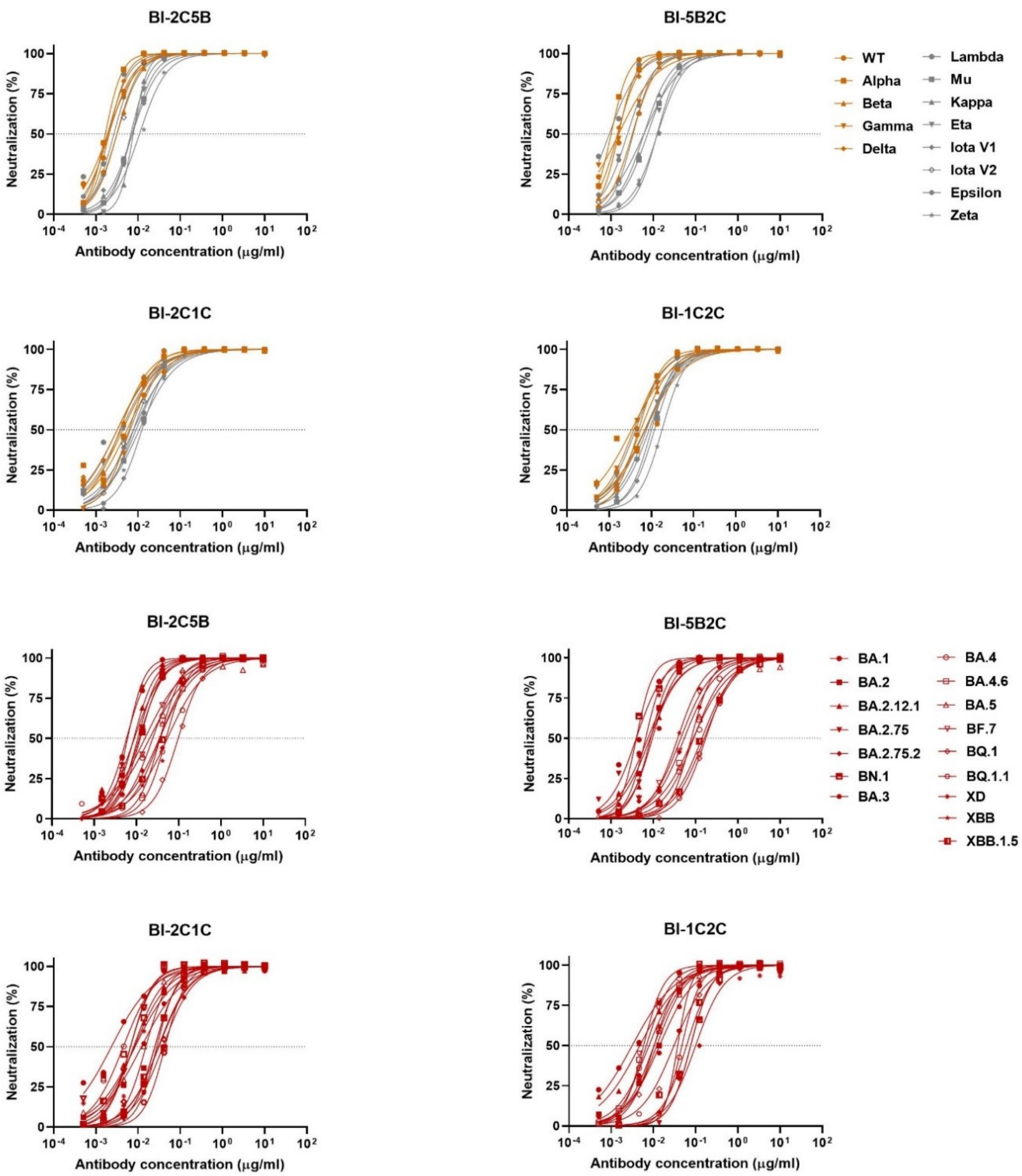

Supplementary Fig. 12 Bispecific antibody neutralization analyzed with SARS-CoV-2 wild-type and variant pseudoviruses. Pseudovirus neutralization assays were performed on human immunodeficiency virus-1 (HIV-1) pseudotyped with the S protein of SARS-CoV-2 wild type and variants using HeLa-hACE2 target cells. The curves were fitted by nonlinear regression (log [inhibitor] vs. normalized response, variable slope). The dashed line indicates a 50% reduction in viral infectivity. Data for each mAb were obtained from one neutralization experiment of duplicate, represented as mean ± s.d.. Individual mAbs were tested independently
